# Lithium: effects in animal models of vanishing white matter are not promising

**DOI:** 10.3389/fnins.2024.1275744

**Published:** 2024-01-30

**Authors:** Diede Witkamp, Ellen Oudejans, Leoni Hoogterp, Gino V. Hu-A-Ng, Kathryn A. Glaittli, Tamara J. Stevenson, Marleen Huijsmans, Truus E. M. Abbink, Marjo S. van der Knaap, Joshua L. Bonkowsky

**Affiliations:** ^1^Child Neurology, Emma Children’s Hospital, Amsterdam Leukodystrophy Center, Amsterdam University Medical Centers, Vrije Universiteit and Amsterdam Neuroscience, Amsterdam, Netherlands; ^2^Department of Integrative Neurophysiology, Center for Neurogenomics and Cognitive Research, VU University, Amsterdam, Netherlands; ^3^Department of Pediatrics, University of Utah, Salt Lake City, UT, United States

**Keywords:** lithium, vanishing white matter, integrated stress response, GSK3**β**, ATF4

## Abstract

Vanishing white matter (VWM) is a devastating autosomal recessive leukodystrophy, resulting in neurological deterioration and premature death, and without curative treatment. Pathogenic hypomorphic variants in subunits of the eukaryotic initiation factor 2B (eIF2B) cause VWM. eIF2B is required for regulating the integrated stress response (ISR), a physiological response to cellular stress. In patients’ central nervous system, reduced eIF2B activity causes deregulation of the ISR. In VWM mouse models, the extent of ISR deregulation correlates with disease severity. One approach to restoring eIF2B activity is by inhibition of GSK3β, a kinase that phosphorylates eIF2B and reduces its activity. Lithium, an inhibitor of GSK3β, is thus expected to stimulate eIF2B activity and ameliorate VWM symptoms. The effects of lithium were tested in zebrafish and mouse VWM models. Lithium improved motor behavior in homozygous *eif2b5* mutant zebrafish. In lithium-treated *2b4^he^2b5^ho^* mutant mice, a paradoxical increase in some ISR transcripts was found. Furthermore, at the dosage tested, lithium induced significant polydipsia in both healthy controls and *2b4^he^2b5^ho^* mutant mice and did not increase the expression of other markers of lithium efficacy. In conclusion, lithium is not a drug of choice for further development in VWM based on the limited or lack of efficacy and significant side-effect profile.

## Introduction

Vanishing white matter (VWM) is leukodystrophy with an autosomal recessive mode of inheritance, which most often presents in young children (Hamilton et al., 2018). This disease causes neurological deterioration characterized by motor decline, cerebellar ataxia, cognitive decline, and premature death; curative treatment is currently not available. White matter pathology is characterized by rarefaction and cystic degeneration with loss of all white matter components. Numbers of immature astrocytes and oligodendrocytes are increased, which fail in their respective mature functions of forming astroglial scar tissue and producing and maintaining sufficient myelin (Bugiani et al., 2011). Astrocyte dysfunction is central to VWM pathogenesis (Dooves et al., 2016).

VWM is caused by bi-allelic pathogenic variants in any of the five genes encoding the subunits (α-ε) of eukaryotic initiation factor 2B (eIF2B) (van der Knaap et al., 2002). The eIF2B protein complex is essential for mRNA translation initiation in all eukaryotic cells due to its function as a guanine nucleotide exchange factor (GEF) for the eIF2 complex (Konieczny and Safer, 1983; Wortham et al., 2014). In addition, eIF2B is central in the integrated stress response (ISR), an adaptive cell response to different types of cellular stress that requires translation attenuation and selective expression of stress-ameliorating proteins (Proud, 2001). eIF2B activity is inhibited via direct phosphorylation of serine 540 in the ε subunit by the glycogen synthase kinase 3β (GSK3β) (Stambolic et al., 1996; Welsh et al., 1998; Beurel et al., 2015) or upon phosphorylation of the conserved serine 51 in the α subunit of eIF2 (eIF2α) (Pakos-Zebrucka et al., 2016). Decreased eIF2B activity directly attenuates bulk protein translation and increases the production of transcription factors ATF4, CHOP, and phosphatase co-factor GADD34 (Lu et al., 2004; Han et al., 2013). ATF4 and CHOP increase the expression of stress-ameliorating proteins (Han et al., 2013), and GADD34 constitutes an important ISR negative feedback loop imperative in eIF2α dephosphorylation and transcription attenuation (Pakos-Zebrucka et al., 2016).

Pathogenic variants in any eIF2B subunit reduce its activity (van Kollenburg et al., 2006; Horzinski et al., 2010; Liu et al., 2011; Wisse et al., 2017; Abbink et al., 2019; Wong et al., 2019). In the brains of VWM patients, reduced eIF2B activity leads to increased ATF4 and CHOP activities and reduced levels of phosphorylated eIF2α (p-eIF2α) (Abbink et al., 2019). This increased ATF4 activity is detected selectively in patients’ astrocytes. eIF2B activators ameliorate neurological disease in VWM mouse models; their degree of disease amelioration correlates strongly with the degree of attenuated expression of ATF4 and its transcriptome (Abbink et al., 2019; Wong et al., 2019). These studies highlight two important findings: (1) eIF2B is a promising treatment target, and (2) the expression levels of ATF4-regulated transcripts are suitable markers for eIF2B activity and VWM pathogenesis.

**Figure 1:**
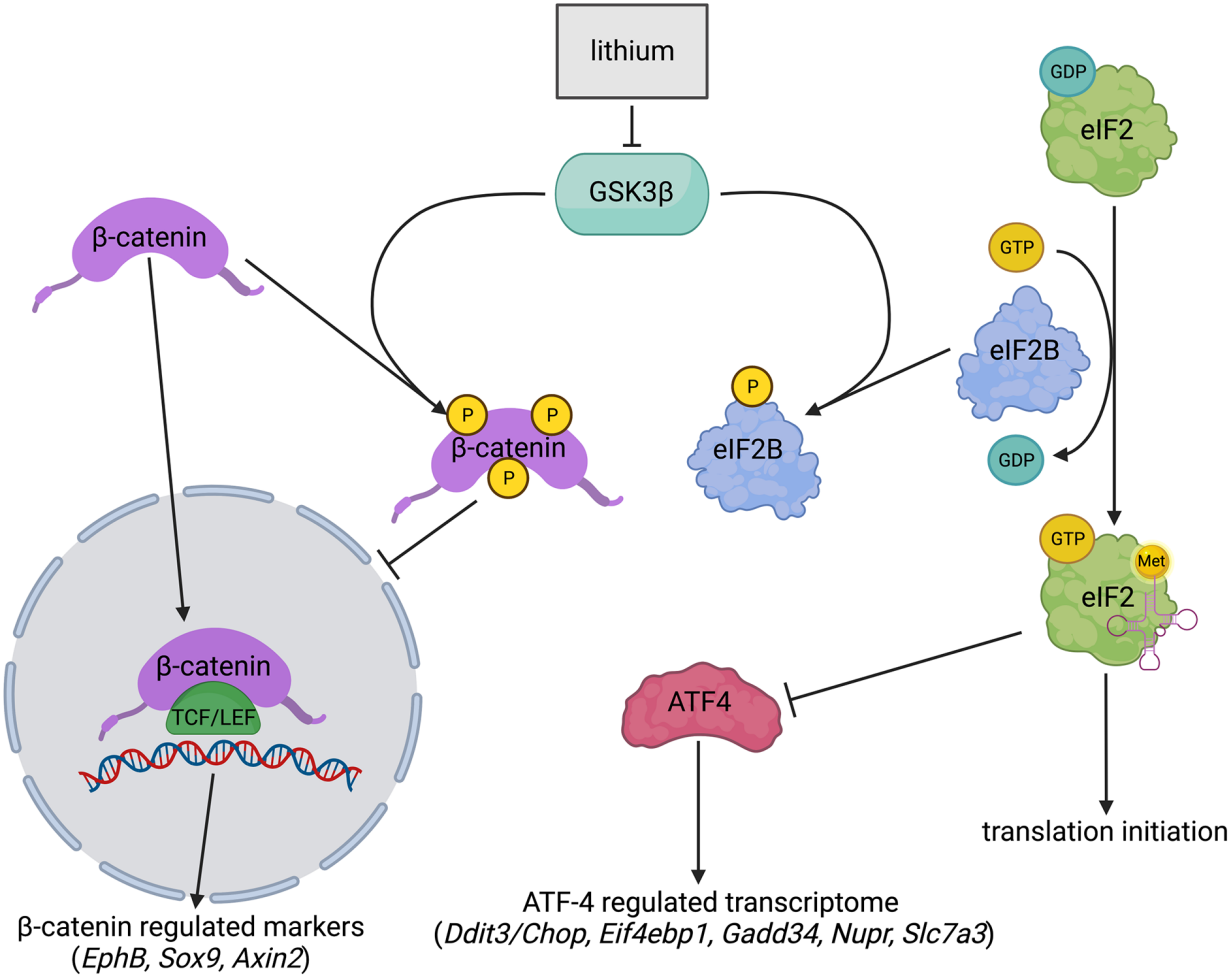
Simplified schematic overview of lithium effects on GSK3β, eIF2B, and β-catenin. Lithium inhibits GSK3β activity. GSK3β inhibition lowers eIF2B phosphorylation (yellow) levels resulting in increased eIF2B activity and presumably downregulated expression of ATF4 and the ATF4-regulated transcriptome. GSK3β inhibition also stabilizes β-catenin by impeding its phosphorylation (yellow) at Ser33, Ser37, and Thr41. Phosphorylated β-catenin is highly unstable and was therefore not quantified. Non-phosphorylated β-catenin moves to the nucleus and joins with a member of the T-cell factor/lymphoid enhancer factor (TCF/LEF) family of transcription factors. This joining upregulates the expression of several genes, including *EphB, Sox9,* and *Axin2*. ➔ indicates stimulation; --| indicates inhibition. The image was created with BioRender.com.

Inhibiting GSK3β to enhance eIF2B activity has been suggested as a potential treatment target for VWM (Halliday et al., 2017; Pavitt, 2018). GSK3β inhibition is expected to enhance eIF2B activity, attenuate the expression of ATF4-regulated transcripts, and ameliorate VWM (Stambolic et al., 1996; Pap and Cooper, 2002; Pavitt, 2005; Pasquali et al., 2010). The transcription-regulating factor β-catenin is another target of GSK3β. GSK3β inhibition increases β-catenin stability, allowing its relocation into the nucleus (Doble and Woodgett, 2003; Valvezan et al., 2012; Stamos and Weis, 2013). Inside the nucleus, β-catenin regulates the expression of specific markers and is part of the Wnt signaling pathway (Figure 1) (Stambolic et al., 1996; Niehrs, 2012). Therefore, GSK3β inhibition is also expected to increase the level of non-phosphorylated β-catenin with consequential increases of β-catenin-regulated transcripts.

Lithium is a GSK3β-inhibiting drug (Stambolic et al., 1996; Beurel et al., 2015), which is used clinically as a treatment for bipolar disorder (Shorter, 2009). In the current project, the potential effect of lithium was assessed in two VWM disease models. Initial studies with lithium were performed in *eif2b5* mutant zebrafish, which is homozygous for an internal deletion in exon 1 and displays reduced locomotor activity, decreased thickness of the myelin sheath, and ISR activation (Keefe et al., 2020). Treatment of these zebrafish resulted in improved motor behavior. Subsequent tests were performed in the *2b4^he^2b5^ho^* mouse model, an established preclinical model for human disease (Abbink et al., 2019; Witkamp et al., 2022). This mouse model replicates clinical and neuropathological signs of human VWM, including the deregulated ISR in the central nervous system (Dooves et al., 2016; Abbink et al., 2019). The levels of ISR-regulated mRNAs in their brain correlate well with disease state and respond quickly to therapeutic targeting of the ISR [e.g., Figure 1 in Witkamp et al. (2022)]. Human eIF2Bε is more conserved in mice than in zebrafish. Due to greater phylogenetic differences between zebrafish and humans than between mice and humans, we decided to validate lithium effects observed in zebrafish in *2b4^he^2b5^ho^* mutant VWM mice before considering application in VWM patients. Lithium-induced side-effects polydipsia and polyuria (Bendz and Aurell, 1999) were monitored. β-catenin and ATF4 activity were investigated by the quantification of the expression of their regulated transcripts.

## Methods

### Animals

#### Zebrafish

Healthy controls (wild-type; WT) and *eif2B5^zc103^* zebrafish were used in this study (Keefe et al., 2020). Heterozygous in-crosses were used for experiments. Genotyping was performed as previously described (Keefe et al., 2020) but consisted of survival genotyping at 72 hpf. In brief, embryos were loaded individually onto genotyping wells of a ZEG chip (wFluidx, Inc.). Once embryos were loaded, the loaded ZEG chip was vibrated. The corresponding embryo was removed from the chip well with a transfer pipette and a small amount of E3 into a 96 square well plate (650 μL/well) until after genotyping. For genotyping PCR, 5 μL of E3 was removed from each ZEG chip well and was used directly in PCR.

All methods have been reported in accordance with recommendations in the ARRIVE guidelines. Adult fish were bred according to standard methods. Embryos were raised at 28.5°C in E3 embryo medium and staged by time and morphology. Zebrafish were randomly allocated to control or experimental groups, and investigators were blinded to genotype and/or experimental status during experiments and analysis of the data. Zebrafish experiments were performed in accordance with the guidelines from the University of Utah Institutional Animal Care and Use Committee (IACUC), regulated under federal law (the Animal Welfare Act and Public Health Services Regulation Act) by the U.S. Department of Agriculture (USDA) and the Office of Laboratory Animal Welfare at the NIH, and accredited by the Association for Assessment and Accreditation of Laboratory Care International (AAALAC). For zebrafish welfare, ethical considerations, and steps to minimize suffering, we followed and used the specific guidelines and regulations at our institution (the University of Utah IACUC, the USDA, and the NIH Office of Laboratory Animal Welfare). Less than 1% of embryos died during the experiment, with no difference between the control and treatment (lithium) groups.

#### Mice

Mouse breeding was done inside the VU-VUmc animal facility. VWM mice heterozygous for eIF2Bδ Arg484Trp and homozygous for eIF2Bε Arg191His mutation (*2b4^he^2b5^ho^*) were used in this study. WT (C57BL/6 J) mice were included as healthy controls. Genotyping of the mutant mouse line was done as before (Dooves et al., 2016), before weaning. The *2b4^he^2b5^ho^* genotype was phenotypically confirmed during weaning on the basis of size. Mice were weaned at P28 and kept with a 12-h light/dark cycle with food and water provided *ad libitum*. Mice were kept in individually ventilated cages and provided with enrichment including a house, nesting material, and gnawing sticks. The humane endpoint (HEP) for the mutant mice was defined by weight loss of more than 15% of body weight for 2 consecutive days or weight loss of more than 20%, although in the current study, HEP was not applied. All mouse experiments were carried out in compliance with the Dutch and European law and with the approval of the local animal care and use committee of the VU University (license CCD AVD1140020172804, work protocol 2,804-NEU19-11). All methods have been reported in accordance with recommendations in the ARRIVE guidelines.

### Lithium treatment in WT and *eif2b5* mutant zebrafish

Collected zebrafish embryos were manually dechorionated at 1 day post-fertilization (dpf) and transferred into the pre-equilibrated solution (E3) containing 1 mM LiCl in water and 0.1% DMSO. The 1 mM concentration is a standard dose and causes inhibition of GSK3β signaling (e.g., Liu et al., 2018). 0.1% DMSO in solution (E3) was used as a placebo control in 50 mL Petri dishes. Embryos were incubated until 7 dpf. Larval behavior analysis consisting of recordings of total movement time, total distance moved, and average velocity was performed on 7 dpf larvae in 96-well square bottom plates (Krackeler Scientific) using automated video analysis software (Noldus EthoVision). Animals were transferred at 6 dpf to the 96-well plate and kept at 28.5°C overnight. At 7 dpf, the plate was placed on the video imaging system, and the animals adapted in the dark for 10 min and recorded for 5 min (1-min dark and 4-min light). Data consisted of 48 WT and 38 *eif2b5^zc103/zc103^* zebrafish treated with placebo and 12 WT and 26 *eif2b5^zc103/zc103^* zebrafish with lithium. In the zebrafish, experiments were performed prior to the biological determination of sex, which happens when zebrafish are older than 2 months.

### Lithium treatment in WT and *2b4^he^2b5^ho^* mutant mice

Lithium chloride (Sigma-Aldrich) was dissolved in water for injection (WFI) (pH ~7.7). Three-month-old WT and *2b4^he^2b5^ho^* mutant VWM female mice were weighed before the start of the experiment and evenly assigned to treatment groups based on this initial body weight to prevent a body weight bias. Other than body weight, the mice were randomly assigned to a treatment group in the presence of two researchers to prevent human error. Two animals of the same genotype and treatment group were housed per cage. The mice were injected daily in the intraperitoneal (i.p.) cavity with 200 mg/kg lithium chloride or placebo (WFI) (*n* = 8 per test condition, *n* = 32 in total). To guarantee constant dosing, we chose i.p. injection over oral gavage, which was considered more burdensome in the very small mice. Can et al. (2011) showed that i.p. injection with this dose resulted in brain lithium concentrations comparable to the human therapeutic range (Can et al., 2011). The duration of the treatment was based on a study from Makoukji et al. (2012) showing beneficial effects on remyelination after nerve-crush injury after 7 days of lithium treatment. The general wellbeing of the animals was monitored throughout the experiment. Researchers performing the injections were not blinded as mouse body weight and lithium-associated side effects would make both genotypes and treatment obvious. Injections were therefore performed in the presence of two researchers to prevent human error. Body weight was measured daily. Lithium-induced polydipsia was monitored by daily weighing of the water bottles. Two empty cages with a water bottle were included as drip control. Water intake was calculated per cage by dividing water intake per 24 h, corrected for general dripping, by the total body weight of the two mice. After 1 week of injections, the mice were euthanized by cervical dislocation 7–8 h after their final injection. This time frame was selected because lithium concentrations in mouse brains were highest between 5 and 8 h after i.p. injection and comparable to the human therapeutic range (Can et al., 2011). Brains were taken out, snap-frozen in liquid nitrogen, and stored at −80°C for molecular examination.

### Quantification of ISR deregulation in mouse brain

The cerebella were prepared for the qPCR and Western blot tests, as these parts of the brain showed amelioration of ISR components in previous studies and have relatively high ratios of white to gray matter (Abbink et al., 2019; Wong et al., 2019; Witkamp et al., 2022). Samples were prepared in the presence of two researchers to minimize human error. qPCR was performed as described (Wisse et al., 2017; Abbink et al., 2018; Witkamp et al., 2022). *Sorcs1* and *Nrxn* mRNA were used as reference genes because the expression of previously used reference genes varied depending on lithium treatment. Oligonucleotide primers for mRNA quantification can be found in Supplementary Data Sheet S2. The researcher performing the qPCR was blinded to treatment and genotype. The SDS-PAGE with 2,2,2-trichloroethanol (TCE) (Ladner et al., 2004) and the Western blotting were done as described (Wisse et al., 2017; Witkamp et al., 2022). Protein yield in one lysate from the lithium-treated VWM group was insufficient for use in the Western blot and was excluded from the study. As primary antibodies, anti-eIF2α SC-11386/SantaCruz (1:1000), anti-phospho-eIF2α (Ser51) 119A11/Cell Signaling (1:1000), and anti-non-phospho (Active) β-Catenin (Ser33/37/Thr41) 8814S/Cell Signaling (1,1,000) were used. HRP-labeled anti-IgG rabbit (1,10,000, Dako, P0448) was used as the secondary antibody. Quantification was done as described (Wisse et al., 2017). Researchers were not blinded to genotype or treatment for the Western blot experiments because the order of samples was set to prevent confounding effects caused by the location of the sample on the blot.

## Statistical analysis

Statistical analyses on zebrafish and mice data were performed with GraphPad Prism 9.3.1 software. Broadly, the statistical analyses follow the statistical analyses that we and others have applied (Kim, 2015; Keefe et al., 2020; Witkamp et al., 2022; Le et al., 2023; Tran et al., 2023; McKnight and Najab, n.d.). Importantly, in certain experiments, WT animals were included to ensure that the disease phenotype in mutant animals was as expected (“the experiment worked”—controls). Once this difference was verified, these control data were removed from the dataset, which was used in subsequent statistical analyses to address the treatment effects in VWM mice. For these subsequent analyses, correction for multiple testing was omitted to minimize the chance of a type 2 error. Differences of *p* <0.05 were considered significant. Statistically significant differences between WT and *eif2b5^zc103/zc103^* placebo-treated zebrafish were examined using an unpaired t-test (Kim, 2015) or Mann–Whitney test (McKnight and Najab, n.d.) (distance moved and velocity; Supplementary Data Sheet S1). Lithium treatment effects were examined separately in WT and *eif2b5^zc103/zc103^* zebrafish using an unpaired t-test or Mann–Whitney test (VWM, distance moved; Supplementary Data Sheet S1). Examination of treatment differences in water intake within and between WT and *2b4^he^2b5^ho^* mutant genotypes was performed with a mixed effects model followed by post-Tukey’s multiple comparison test. For results obtained using qPCR and the Western blot, session variation was corrected with the software program factor (Ruijter et al., 2006). Variation in treatments or conditions was not corrected in this program. For qPCR, the mean Cp values of the reference genes *Sorcs1* and *Nrxn* were calculated per sample using the LightCycler 480-II software as described in Wisse et al. (2017). Relative expression of the target gene was obtained by dividing the Cp value of the target gene with the mean Cp value of the two reference genes (Livak and Schmittgen, 2001; Pfaffl, 2001; Schmittgen and Livak, 2008; Riedel et al., 2014). For the Western blots, protein signal values were corrected over TCE values (loading control) per lane. Relative levels of phosphorylated eIF2α were determined by dividing their signals with those of total eIF2α (Harding et al., 2000b). Statistically significant differences between WT and *2b4^he^2b5^ho^* mutant placebo-treated mice were examined using an unpaired t-test or Mann–Whitney test (relative *EphB, Sox9, Axin2,* eIF2α phosphorylation, *Ddit3/Chop, Eif4ebp1, Gadd34, Nupr, Trib3, and Slc3a2* levels) (Supplementary Data Sheet S1). If a statistically significant difference was observed, lithium treatment effects were examined separately in WT and *2b4^he^2b5^ho^* mutant mice using an unpaired t-test or Mann–Whitney test (VWM: relative *Ddit3/Chop*, *Gadd34*, *Nupr, Psat1* levels) (Supplementary Data Sheet S1). When there was no significant difference between the WT and VWM phenotypes, data were analyzed using a two-way ANOVA (Gelman, 2005) (Supplementary Data Sheet S1).

## Results

### Lithium ameliorates velocity in *eif2B5^zc103^* mutant zebrafish

**Figure 2:**
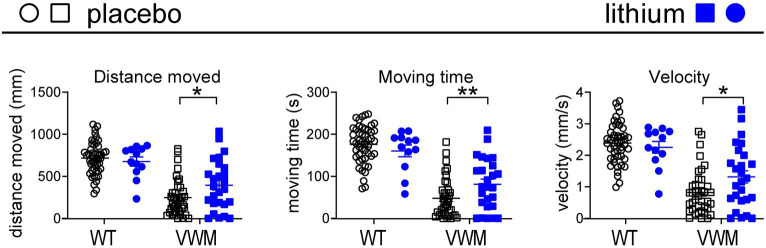
Lithium improves motor deficits in mutant *eif2b5^zc103/zc103^* zebrafish. WT (circles) and *eif2b5^zc103/zc103^* (VWM; squares) zebrafish were treated with placebo (black; WT: *n* = 48, VWM*: n* = 38) or 1 mM lithium (blue; WT: *n* = 12, VWM: *n* = 26) and assessed on distance moved, moving time, and velocity at 7 dpf. The graphs show individual and mean values, +/− SEM. The shown motor behavior parameters differ significantly in placebo-treated WT versus placebo-treated *eif2b5^zc103/zc103^* zebrafish (*p* < 0.0001; not indicated, Supplementary Data Sheet S1). Treatment effects were statistically analyzed using an unpaired *t*-test or Mann–Whitney test (VWM, distance moved; Supplementary Data Sheet S1). *p < 0.05 and **p < 0.01.

To test the effects of lithium on VWM, a previously published zebrafish (*Danio rerio*) small vertebrate model of VWM (Keefe et al., 2020) was used. Zebrafish *eif2B5^zc103^* homozygous mutants show impaired swimming behavior: their distance moved, time, and swimming velocity were, respectively, −66, −73%, and − 66% lower than in wild-type (WT) fish (all *p* < 0.0001; Figure 2). Lithium or placebo was administered to WT (placebo: *n* = 48, lithium: *n* = 12) or *eif2B5^zc103^* mutant zebrafish (placebo: *n* = 38, lithium: *n* = 26), and their swimming velocity was determined by assessing distance and time. Lithium did not affect distance moved, time, or swimming velocity in WT zebrafish but significantly improved all these parameters in mutant zebrafish (+60%, *p* = 0.0202; +70%, *p* = 0.0079; +60%, *p* = 0.0120, respectively; Figure 2; Supplementary Data Sheet S1).

### Lithium increases water intake in WT and in *2b4^he^2b5^ho^* mutant mice

**Figure 3:**
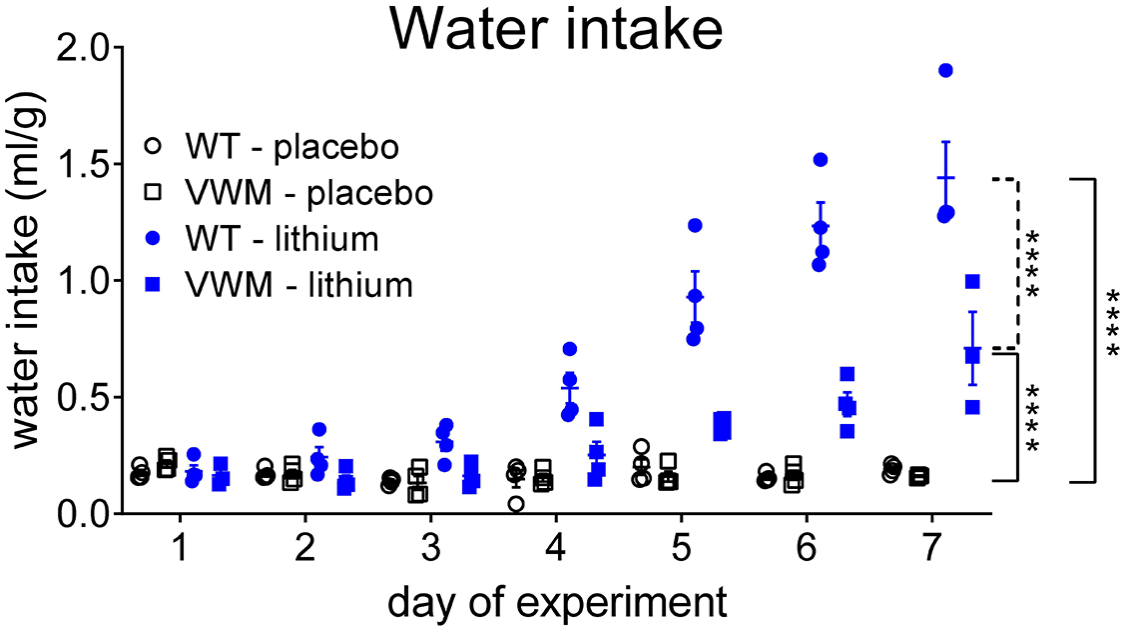
Lithium induces more pronounced polydipsia in WT than in *2b4^he^2b5^ho^* mutant mice. WT (circles) and *2b4^he^2b5^ho^* mutant (VWM; squares) mice were i.p. injected with placebo (black) or 200 mg/kg lithium (blue) (*n* = 8 per treatment, per genotype). Water intake (ml/g) is shown per cage housing two mice (individual and mean values, +/− SEM). Statistical analysis was performed with a mixed-effects model with *post-hoc* Tukey’s multiple comparison test (Supplementary Data Sheet S1). ****p < 0.0001; placebo-lithium differences: ^____^, WT-VWM difference: ----. Raw data are in Supplementary Data Sheet S2.

Lithium increased water intake in WT and *2b4^he^2b5^ho^* mutant mice and was accompanied by markedly increased urination, as observed by the wetness of the cage bedding. The start and extent of the response to lithium injections differed per genotype (*n* = 8 per treatment, per genotype) (interaction effect time x treatment x genotype: *F*(6, 70) = 12,68, *p* < 0.0001). The lithium-induced increased water intake was significantly higher in WT mice than in *2b4^he^2b5^ho^* mutant mice (*p* < 0.0001). The water intake increase was also earlier in WT mice: Lithium-injected WT mice started to drink more from experimental day 4 onward (+260%, *p* = 0.0006) but in *2b4^he^2b5^ho^* mutant mice not until experimental day 6 (+185%, *p* = 0.0298) as compared to their placebo-treated genotype controls. Lithium injections increased water intake most on the final day of the experiment in both WT (day 7: +651%, *p* < 0.0001) and *2b4^he^2b5^ho^* mutant mice (day 7: +342%, *p* < 0.0001) as compared to placebo-treated mice (Figure 3; Supplementary Data Sheet S1).

### Lithium does not increase the levels of β-catenin or its regulated mRNAs in mouse cerebellum

**Figure 4:**
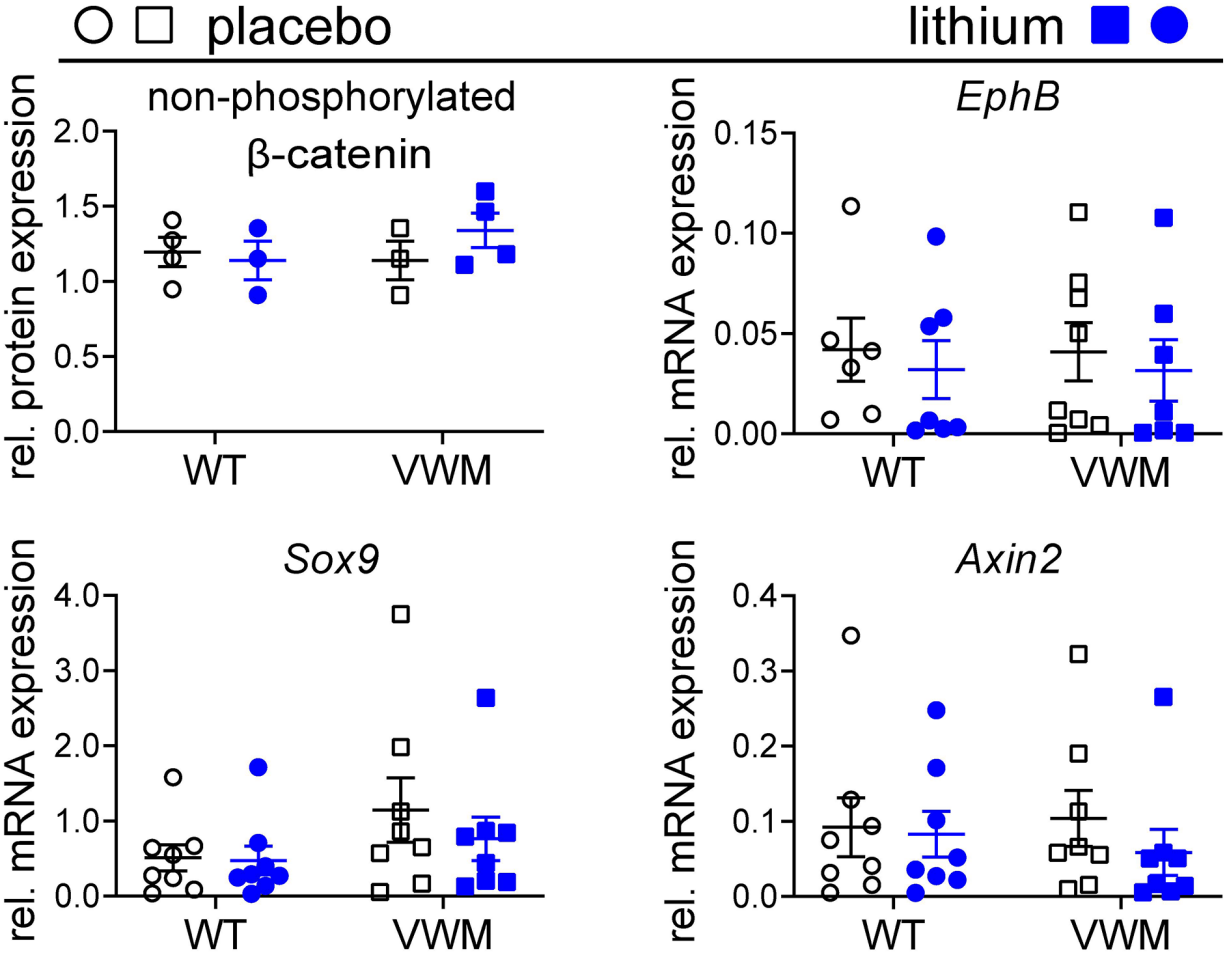
Lithium does not increase the expression and activity of non-phosphorylated β-catenin. WT (circles) and *2b4^he^2b5^ho^* mutant (VWM; squares) mice (*n* = 8 per treatment, per genotype) were i.p. injected daily with placebo (black) or 200 mg/kg lithium (blue) for 1 week. Mice were terminated approximately 8 h after the final injection. Cerebellar RNA and protein samples were derived from the same lysate. Levels of non-phosphorylated β-catenin were determined using the Western blot (*n* = 3–4). Lithium treatment effects on indicated β-catenin-regulated markers were determined with qPCR (*n* = 6–8) (*Sorcs1* and *Nrxn* as reference). Lithium differences were analyzed with two-way ANOVAs (Supplementary Data Sheet S1). The graphs show individual and mean values, +/− SEM. Raw data are available in Supplementary Data Sheet S2.

Lithium is reported to increase the stability and transcription-regulating activity of β-catenin (O’brien et al., 2004), which increases transcription of β-catenin-regulated mRNAs *EphB* (Batlle et al., 2002; Sansom et al., 2004)*, Sox9* (Blache et al., 2004; Panza et al., 2013), and *Axin2* (Jho et al., 2002; Clevers, 2006; Fancy et al., 2011) in mouse brain. Baseline expression of β-catenin and its downstream targets was similar in the brains of placebo-treated WT and *2b4^he^2b5^ho^* mutant mice (Figure 4). Here, we find that lithium did not substantially or consistently affect the accumulation of non-phosphorylated β-catenin (WT: −5%, *p* = 0.9312, *2b4^he^2b5^ho^* mutant: +18%, *p* = 0.4446) and its regulated mRNAs in the brain (Figure 4; Supplementary Data Sheet S1; Supplementary Figure S2).

### Lithium enhances the deregulated expression of some ATF4-regulated targets

**Figure 5:**
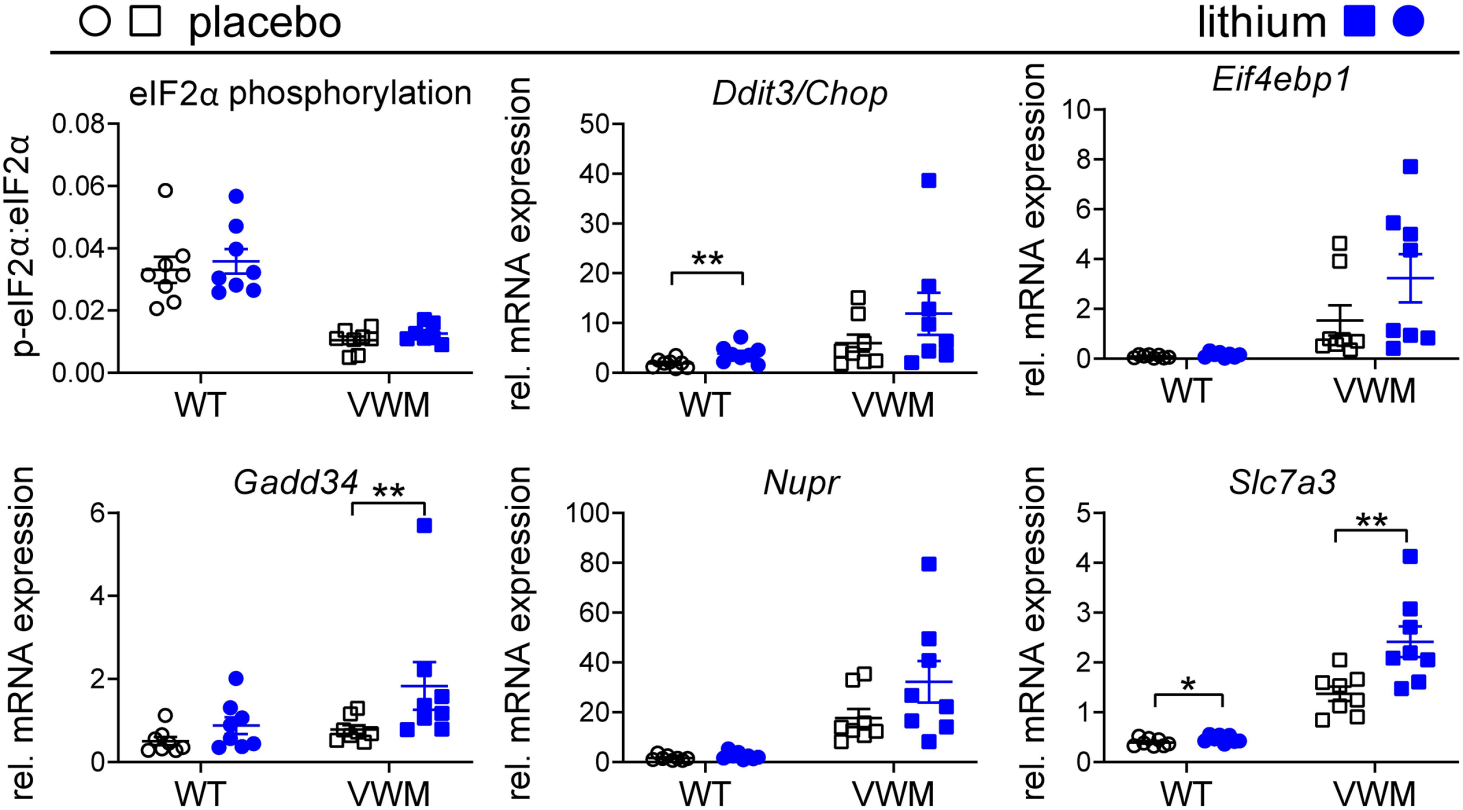
Lithium subtly increases the expression of the ATF4-regulated transcriptome in the mouse brain. WT (circles) and *2b4^he^2b5^ho^* mutant (VWM; squares) mice were i.p. injected daily with 200 mg/kg lithium (blue) or placebo (black) for 1 week (*n* = 8 per treatment, per genotype). Mice were terminated approximately 8 h after injection. Cerebellar RNA and protein samples were derived from the same lysate. Lithium effects on indicated ISR markers were determined using the Western blot for eIF2α phosphorylation (*n* = 7–8) and qPCR for mRNA expression (*Sorcs1* and *Nrxn* as reference, *n* = 8). The graphs show individual and mean values, +/− SEM. The shown ISR markers differ significantly in placebo-treated WT versus placebo-treated *2b4^he^2b5^ho^* mice (*p* < 0.05; not indicated, Supplementary Data Sheet S1). Treatment effects were statistically analyzed using an unpaired *t*-test or Mann–Whitney test (VWM: *Ddit3/Chop*, *Gadd34*, *Nupr*; Supplementary Data Sheet S1). Raw data are available in Supplementary Data Sheet S2. *p < 0.05 and **p < 0.01.

Lithium-dependent effects on the ISR were investigated as a measure of ATF4 activity. eIF2α phosphorylation levels were lower, and ATF4-regulated mRNA levels were higher in the *2b4^he^2b5^ho^* mutant than in the WT mouse brain (Abbink et al., 2019). Remarkably, lithium treatment somewhat increased eIF2α phosphorylation that was not statistically significant in the WT (+8%, *p* = 0.3193) or *2b4^he^2b5^ho^* mutant mice (+21%, *p* = 0.1087) relative to the placebo treatment. Unexpectedly, the expression of ATF4-regulated mRNAs was higher in the lithium-treated than in placebo-treated *2b4^he^2b5^ho^* mutant mice, although statistical significance was reached only for some (Figure 5; Supplementary Figures S1, S3; Supplementary Data Sheet S1). mRNA expression levels of *Gadd34*, *Nupr*, and *Slc7a3* were notable for being increased by lithium treatment (+81%, *p* = 0.0035; +84%, *p* = 0.0524; +76%, *p* = 0.0041, respectively). A similar effect was observed in lithium-injected WT mice, in which statistical significance was reached for the lithium-induced *Ddit3*/*Chop* and *Slc7a3* mRNA expression (+101%, *p* = 0.0071 and + 17%, *p* = 0.0403, respectively), and a statistical trend was observed for *Gadd34* mRNA expression (+74%, *p* = 0.061).

## Discussion

Mutant *eif2b5* zebrafish and *2b4^he^2b5^ho^* mice were subjected to lithium treatments, which were expected to inhibit GSK3β and consequently enhance eIF2B activity, resulting in improved disease signs. Mutant *eif2b5* zebrafish displayed improved swimming behavior in response to lithium treatment. Lithium treatment induced the known side effects of polydipsia and polyuria in the WT and *2b4^he^2b5^ho^* mutant mice indicating drug-induced nephrogenic diabetes insipidus (Bendz and Aurell, 1999). The lithium treatment regimen did, however, not show any clinical beneficial effects in *2b4^he^2b5^ho^* mutant mice. The side effects limited our ability to increase the dose of lithium and prevented us from fully testing the efficacy of lithium in the VWM mice.

Curiously and contrary to our initial hypothesis and an earlier study (Sun et al., 2014), the brain tissue from the lithium-treated mice showed a somewhat enhanced expression of several ATF4-regulated transcripts. These transcript levels were affected by lithium in both WT and *2b4^he^2b5^ho^* mutant mouse brains, although the effects differed for individual transcripts between genotypes. As ATF4 translation increases in response to reduced eIF2B activity (Harding et al., 2000a; Young and Wek, 2016), upregulation of ATF4-regulated targets suggests that lithium increased ATF4 translation and reduced eIF2B activity even further. Statistical analyses were hampered by the high degree of variation, especially in the lithium-treated VWM mice, obscuring the clear effects of lithium. Still, in the *2b4^he^2b5^ho^* mutant mouse brains, ATF4-regulated targets were consistently upregulated by lithium. It is unlikely that the effect of lithium on the ATF4-regulated transcripts of astrocytes is masked by the effects on other cell types present in the brain tissue/cerebellum, such as oligodendrocytes, because the ATF4 and ATF4-regulated mRNAs are only detected in VWM astrocytes (Abbink et al., 2019). Lithium was not effective in increasing non-phosphorylated β-catenin protein levels in either WT or *2b4^he^2b5^ho^* mutant mouse brain, which is a main effect downstream of GSK3β inhibition (Wu and Pan, 2010). In line with this negative finding and similar to other studies (Leroy et al., 2010; Del Grosso et al., 2022), increased lithium-induced β-catenin activity in the brain was not observed, and the expression of β-catenin-regulated mRNAs in the WT and *2b4^he^2b5^ho^* mutant mouse brain was unaltered by lithium. The unchanged β-catenin activity in the brain suggests that GSK3β inhibition by lithium was negligible or temporary. Therefore, it remains an open question if the altered ATF4 expression in the *2b4^he^2b5^ho^* mutant mouse brain was induced via lithium-dependent GSK3β inhibition or via an alternative route.

Limitations of this study include that lithium impacts multiple biochemical pathways, making the determination of the precise mechanism of action less certain. In addition, we were limited in assessing higher dosages of lithium in mice because of the observed toxicities. Although we did not measure lithium concentration in the brain or blood, the observed toxicities and increase in ATF4 activity show that lithium was active in the animals. Our experimental approach was to use zebrafish as a rapid and inexpensive screening platform, with subsequent validation and characterization in an established mouse model, representative of VWM.

The effects of lithium in the mutant *eif2b5* zebrafish and the *2b4^he^2b5^ho^*mouse model perhaps differ. In both animal models, the catalytic subunit eIF2Bε, the target of GSK3β, is mutant, although the exact mutation differs between the animal models. Further tests are needed to investigate whether the mutant eIF2B complex in the mutant *eif2b5* zebrafish and *2b5^he^2b5^ho^* mice is similarly under normal control of GSK3β activity. In addition, lithium effects were studied in female mice as a homogenous experimental group to reduce the number of animals needed for the experiment. To the best of our knowledge, sex-related differences in lithium brain concentrations or therapeutic effects have not been reported, suggesting that they are similar in VWM female and male mice (Pizzasegola et al., 2009; Can et al., 2011).

Lithium is a drug with a multitude of effects on many proteins and pathways (Hart, 1979; Roux and Dosseto, 2017). Possibly, lithium’s inhibitory effects on eIF2B activity occur via a GSK3β-independent mechanism. Lithium may have caused dehydration (Bendz and Aurell, 1999), which has been described to activate ATF4 (Greenwood et al., 2015). WT mice experienced more lithium-induced polydipsia than *2b4^he^2b5^ho^* mutant mice. However, the levels of ATF4-regulated mRNAs increased more in the *2b4^he^2b5^ho^* mutant than in the WT brain, and lithium had no impact on weight, arguing against dehydration. Another possible explanation for the negative results in mice is that the given dosage of 200 mg/kg lithium was not high enough to inhibit GSK3β in the mouse brains. In this study, higher lithium dosages were not pursued for three reasons. First, higher dosages may not be more effective as a lithium dosage of 200 mg/kg has been shown to be equally effective in antidepressant-like responses as compared to higher dosages in C57BL/6 J mice. Second, the current dosage already induced severe polydipsia. If a higher lithium dosage would be necessary for beneficial ISR effects, then translation to human VWM patients would most likely be difficult. VWM mainly presents in young children (Hamilton et al., 2018), and lithium has reported additional side effects in pediatric patients with bipolar disorders (Rosen, 2017). Third, lithium treatment actually increased the disease-associated ISR deregulation in the *2b4^he^2b5^ho^* mutant mice, suggesting a potential risk for increasing the disease severity (Abbink et al., 2019; Wong et al., 2019). A recent study confirmed the idea that treatment with a high lithium dosage has deteriorating effects on motor skills (Li et al., 2020). Reducing the dosage would limit the observed toxicities but would not translate to the human therapeutic range and would be less likely to inhibit GSK3β than the current dosage of 200 mg/kg (Can et al., 2011). Considering lithium’s already well described and sometimes serious side effects in bipolar patients (Volkmann et al., 2020), the apparent lack of efficacy, and the evidence of potential ISR-activating action in *2b4^he^2b5^ho^* mutant mice, it is not a drug of choice for further development in VWM patients.

Further research is needed to establish if GSK3β inhibition is a suitable strategy for attenuating the deregulated ISR in VWM. Highly specific and potent GSK3β inhibitors that cross the blood–brain barrier may warrant more studies and are expected to have less side effects than lithium (Meares et al., 2011). For example, the antidepressant trazodone has ISR-modulating properties, possibly by inhibiting GSK3β (Halliday et al., 2017; Pavitt, 2018). Other strategies for future investigations could include the use of antisense oligonucleotides to downregulate GSK3β expression including with specific CNS targeting by using intrathecal injections. In conclusion, the data show that lithium may not be a good therapeutic option but that GSK3β remains a target of interest to be investigated for VWM.

## Data availability statement

The original contributions presented in the study are included in the article/Supplementary material, further inquiries can be directed to the corresponding author.

## Ethics statement

Zebrafish experiments were performed in accordance of guidelines from the University of Utah Institutional Animal Care and Use Committee (IACUC), regulated under federal law (the Animal Welfare Act and Public Health Services Regulation Act) by the U.S. Department of Agriculture (USDA) and the Office of Laboratory Animal Welfare at the NIH, and accredited by the Association for Assessment and Accreditation of Laboratory Care International (AAALAC). All mouse experiments were carried out in compliance with the Dutch and European law and with approval of the local animal care and use committee of the VU University (license CCD AVD1140020172804, work protocol 2,804-NEU19-11). The study was conducted in accordance with the local legislation and institutional requirements.

## Author contributions

DW: Formal analysis, Investigation, Methodology, Project administration, Visualization, Writing – original draft, Writing – review & editing. EO: Investigation, Methodology, Writing – review & editing. LH: Investigation, Methodology, Writing – review & editing. GH-A-N: Investigation, Methodology, Writing – review & editing. KG: Investigation, Methodology, Writing – review & editing. TS: Investigation, Methodology, Writing – review & editing. MH: Investigation, Methodology, Writing – review & editing. TA: Investigation, Methodology, Project administration, Supervision, Writing – original draft, Writing – review & editing. MK: Conceptualization, Funding acquisition, Supervision, Writing – review & editing. JB: Conceptualization, Funding acquisition, Investigation, Methodology, Supervision, Visualization, Writing – original draft, Writing – review & editing.

## Supplementary material

SUPPLEMENTARY DATA SHEET 1 Statisitcal analysis.

SUPPLEMENTARY DATA SHEET 2 qPCR primers.

SUPPLEMENTARY TABLE 1 Raw data.

SUPPLEMENTARY IMAGE 1 Subtle increase of the ATF4-regulated transcriptome in the mouse cerebellum by lithium.

SUPPLEMENTARY IMAGE 2 Overview of gels and immunoblots used for the quantification of non-phosphorylated beta-catenin shown in figure 4.

SUPPLEMENTARY IMAGE 3 Overview of gels and immunoblots used for the quantification of eIF2α phosphorylation shown in figure 5.
