# Supplementary material for: Lithium: effects in animal models of vanishing white matter are not promising
Source: Front Neurosci. 2024 Jan 30;18:1275744. doi: 10.3389/fnins.2024.1275744 (PMC10861708; doi:10.3389/fnins.2024.1275744)
Supplement: SUPPLEMENTARY DATA SHEET 1 — Statisitcal analysis. [file Data_Sheet_1.PDF]

Lithium treatment in animal models of vanishing white matter

Diede Witkamp[1], Ellen Oudejans[1], Leoni Hoogterp[1], Gino V. Hu-A-Ng[1], Kathryn A. Glaittli[2], Tamara J. Stevenson[2], Marleen Huijsmans[1], Truus E.M. Abbink[1#], Marjo S. van der Knaap[1#*], Joshua L. Bonkowsky[2#]

Figure 2

**Table Analyzed**	**WT - plac vs VWM - plac Distance moved**		**Basic test parameter: WT-VWM differences**
Mann Whitney test			
P value	<0,0001		
Exact or approximate P value?	Exact		
P value summary	****		
One- or two-tailed P value?	One-tailed		
Sum of ranks in column A,B	2870 , 871		
Mann-Whitney U		130	
Median WT	725,4, n=48		
Median VWM	193,9, n=38		
Difference: Actual		-531,4	
Difference: Hodges-Lehmann		-498,9	
**Table Analyzed**	**WT treatment- Distance moved**		**Basic test parameter: Treatment effect in WT animals**
Unpaired t test			
P value		0,2568	
P value summary	ns		
One- or two-tailed P value?	One-tailed		
t, df	t=0,6572, df=58		
Mean placebo		716,4	
Mean lithium		675,9	
95% confidence interval	-163,7 to 82,76		
**Table Analyzed**	**VWM treatment Distance moved**		**Treatment effects in VWM animals**
Mann Whitney test			
P value		0,0202	
Exact or approximate P value?	Exact		
P value summary	*		
One- or two-tailed P value?	One-tailed		
Sum of ranks in column A,B	1085 , 995		
Mann-Whitney U		344	
Median placebo	193,9, n=38		
Median lithium	364,5, n=26		
Difference: Actual		170,6	
Difference: Hodges-Lehmann		135,7	
**Table Analyzed**	**WT- plac vs VWM-plac Moving time**		**Basic test parameter: WT-VWM differences**
Unpaired t test			
P value	<0,0001		
P value summary	****		
One- or two-tailed P value?	One-tailed		
t, df	t=13,01, df=84		
Mean placebo		176,1	
Mean lithium		47,86	
95% confidence interval	-147,8 to -108,6		
**Table Analyzed**	**WT treatment- Moving time**		**Basic test parameter: Treatment effect in WT animals**
Unpaired t test			
P value		0,1445	
P value summary	ns		
One- or two-tailed P value?	One-tailed		
t, df	t=1,070, df=58		
Mean placebo		176,1	
Mean lithium		160,4	
95% confidence interval	-44,95 to 13,63		
**Table Analyzed**	**VWM treatment - Moving time**		**Treatment effects in VWM animals**
Unpaired t test			
P value		0,0079	
P value summary	**		
One- or two-tailed P value?	One-tailed		
t, df	t=2,480, df=62		
Mean placebo		47,86	
Mean lithium		81,13	
95% confidence interval	6,453 to 60,10		
**Table Analyzed**	**WT-plac VWM-plac Velocity**		**Basic test parameter: WT-VWM differences**
Mann Whitney test			
P value	<0,0001		
Exact or approximate P value?	Exact		
P value summary	****		
One- or two-tailed P value?	One-tailed		
Sum of ranks in column A,B	2870 , 871		
Mann-Whitney U		130	
Median placebo	2,420, n=48		
Median lithium	0,6468, n=38		
Difference: Actual		-1,773	
Difference: Hodges-Lehmann		-1,664	
**Table Analyzed**	**WT treatment - Velocity**		**Basic test parameter: Treatment effect in WT animals**
Unpaired t test			
P value		0,2565	
P value summary	ns		
One- or two-tailed P value?	One-tailed		
t, df	t=0,6581, df=58		
Mean placebo		2,389	
Mean lithium		2,254	
95% confidence interval	-0,5460 to 0,2758		
**Table Analyzed**	**VWM treatment - Velocity**		**Treatment effects in VWM animals**
Unpaired t test			
P value		0,012	
P value summary	*		
One- or two-tailed P value?	One-tailed		
t, df	t=2,315, df=62		
Mean placebo		0,8231	
Mean lithium		1,317	
95% confidence interval	0,06736 to 0,9195		

Figure 3

**Table Analyzed**	**Water/weight**			
Mixed-effects model (REML)	Matching by factor: Time			
Assume sphericity?	Yes			
Alpha	0,05			
Fixed effects (type III)	P value	P value summary	Statistically significant (P < 0,05)?	F (DFn, DFd)
Time	<0,0001	****	Yes	F (6, 70) = 76,45
Treatment	<0,0001	****	Yes	F (1, 12) = 78,38
Genotype	0,0004	***	Yes	F (1, 12) = 23,70
Time x Treatment	<0,0001	****	Yes	F (6, 70) = 74,36
Time x Genotype	<0,0001	****	Yes	F (6, 70) = 15,14
Treatment x Genotype	0,0005	***	Yes	F (1, 12) = 22,79
Time x Treatment x Genotype	<0,0001	****	Yes	F (6, 70) = 12,68
Random effects	SD	Variance		
Subject	0,07108	0,005052		
Residual	0,08038	0,006461		
Was the matching effective?				
Chi-square, df	25,46, 1			
P value	<0,0001			
P value summary	****			
Is there significant matching (P < 0.05)?	Yes			
Data summary				
Number of columns	2 x 2			
Number of rows (Time)	7			
Number of subjects (Subject)	16			
Number of missing values	2			

Compare each cell mean with every other cell mean

Number of families	1
Number of comparisons per family	378
Alpha	0,05

Tukey's multiple comparisons test	Predicted (LS) mean diff,	95,00% CI of diff,	Below threshold?	Summary	Adjusted P Value
1:WT - placebo vs. 1:$2b4^{he}2b5^{ho}$ - placebo	-0,03916	-0,3311 to 0,2528	No	ns	>0,9999
1:WT - placebo vs. 1:WT - lithium	-0,00706	-0,2990 to 0,2849	No	ns	>0,9999
1:WT - placebo vs. 1:$2b4^{he}2b5^{ho}$ - lithium	0,0317	-0,2754 to 0,3388	No	ns	>0,9999
1:WT - placebo vs. 2:WT - placebo	0,004728	-0,2153 to 0,2248	No	ns	>0,9999
1:WT - placebo vs. 2:$2b4^{he}2b5^{ho}$ - placebo	0,005151	-0,2868 to 0,2971	No	ns	>0,9999
1:WT - placebo vs. 2:WT - lithium	-0,06829	-0,3603 to 0,2237	No	ns	>0,9999
1:WT - placebo vs. 2:$2b4^{he}2b5^{ho}$ - lithium	0,03126	-0,2607 to 0,3232	No	ns	>0,9999
1:WT - placebo vs. 3:WT - placebo	0,03567	-0,1844 to 0,2557	No	ns	>0,9999
1:WT - placebo vs. 3:$2b4^{he}2b5^{ho}$ - placebo	0,04344	-0,2485 to 0,3354	No	ns	>0,9999
1:WT - placebo vs. 3:WT - lithium	-0,1327	-0,4247 to 0,1592	No	ns	0,9927
1:WT - placebo vs. 3:$2b4^{he}2b5^{ho}$ - lithium	0,01255	-0,2794 to 0,3045	No	ns	>0,9999
1:WT - placebo vs. 4:WT - placebo	0,02595	-0,1941 to 0,2460	No	ns	>0,9999
1:WT - placebo vs. 4:$2b4^{he}2b5^{ho}$ - placebo	0,02113	-0,2708 to 0,3131	No	ns	>0,9999
1:WT - placebo vs. 4:WT - lithium	-0,3632	-0,6551 to -0,07120	Yes	**	0,0022
1:WT - placebo vs. 4:$2b4^{he}2b5^{ho}$ - lithium	-0,07681	-0,3688 to 0,2152	No	ns	>0,9999
1:WT - placebo vs. 5:WT - placebo	-0,02547	-0,2455 to 0,1946	No	ns	>0,9999
1:WT - placebo vs. 5:$2b4^{he}2b5^{ho}$ - placebo	0,01536	-0,2766 to 0,3073	No	ns	>0,9999
1:WT - placebo vs. 5:WT - lithium	-0,7537	-1,046 to -0,4618	Yes	****	<0,0001
1:WT - placebo vs. 5:$2b4^{he}2b5^{ho}$ - lithium	-0,2016	-0,4936 to 0,09035	No	ns	0,6282
1:WT - placebo vs. 6:WT - placebo	0,0201	-0,1999 to 0,2401	No	ns	>0,9999
1:WT - placebo vs. 6:$2b4^{he}2b5^{ho}$ - placebo	0,01022	-0,2818 to 0,3022	No	ns	>0,9999
1:WT - placebo vs. 6:WT - lithium	-1,059	-1,351 to -0,7669	Yes	****	<0,0001
1:WT - placebo vs. 6:$2b4^{he}2b5^{ho}$ - lithium	-0,2948	-0,5868 to -0,002868	Yes	*	0,0448
1:WT - placebo vs. 7:WT - placebo	-0,01668	-0,2367 to 0,2034	No	ns	>0,9999
1:WT - placebo vs. 7:$2b4^{he}2b5^{ho}$ - placebo	0,0139	-0,2781 to 0,3059	No	ns	>0,9999
1:WT - placebo vs. 7:WT - lithium	-1,266	-1,558 to -0,9740	Yes	****	<0,0001
1:WT - placebo vs. 7:$2b4^{he}2b5^{ho}$ - lithium	-0,5382	-0,8453 to -0,2310	Yes	****	<0,0001
1:$2b4^{he}2b5^{ho}$ - placebo vs. 1:WT - lithium	0,0321	-0,2599 to 0,3241	No	ns	>0,9999
1:$2b4^{he}2b5^{ho}$ - placebo vs. 1:$2b4^{he}2b5^{ho}$ - lithium	0,07086	-0,2363 to 0,3780	No	ns	>0,9999
1:$2b4^{he}2b5^{ho}$ - placebo vs. 2:WT - placebo	0,04389	-0,2481 to 0,3359	No	ns	>0,9999
1:$2b4^{he}2b5^{ho}$ - placebo vs. 2:$2b4^{he}2b5^{ho}$ - placebo	0,04431	-0,1757 to 0,2643	No	ns	>0,9999
1:$2b4^{he}2b5^{ho}$ - placebo vs. 2:WT - lithium	-0,02913	-0,3211 to 0,2628	No	ns	>0,9999
1:$2b4^{he}2b5^{ho}$ - placebo vs. 2:$2b4^{he}2b5^{ho}$ - lithium	0,07042	-0,2215 to 0,3624	No	ns	>0,9999
1:$2b4^{he}2b5^{ho}$ - placebo vs. 3:WT - placebo	0,07483	-0,2171 to 0,3668	No	ns	>0,9999
1:$2b4^{he}2b5^{ho}$ - placebo vs. 3:$2b4^{he}2b5^{ho}$ - placebo	0,0826	-0,1374 to 0,3026	No	ns	0,9995
1:$2b4^{he}2b5^{ho}$ - placebo vs. 3:WT - lithium	-0,09358	-0,3856 to 0,1984	No	ns	>0,9999
1:$2b4^{he}2b5^{ho}$ - placebo vs. 3:$2b4^{he}2b5^{ho}$ - lithium	0,05172	-0,2403 to 0,3437	No	ns	>0,9999
1:$2b4^{he}2b5^{ho}$ - placebo vs. 4:WT - placebo	0,06511	-0,2269 to 0,3571	No	ns	>0,9999
1:$2b4^{he}2b5^{ho}$ - placebo vs. 4:$2b4^{he}2b5^{ho}$ - placebo	0,0603	-0,1597 to 0,2803	No	ns	>0,9999
1:$2b4^{he}2b5^{ho}$ - placebo vs. 4:WT - lithium	-0,324	-0,6160 to -0,03204	Yes	*	0,0134
1:$2b4^{he}2b5^{ho}$ - placebo vs. 4:$2b4^{he}2b5^{ho}$ - lithium	-0,03764	-0,3296 to 0,2543	No	ns	>0,9999
1:$2b4^{he}2b5^{ho}$ - placebo vs. 5:WT - placebo	0,01369	-0,2783 to 0,3057	No	ns	>0,9999
1:$2b4^{he}2b5^{ho}$ - placebo vs. 5:$2b4^{he}2b5^{ho}$ - placebo	0,05452	-0,1655 to 0,2746	No	ns	>0,9999
1:$2b4^{he}2b5^{ho}$ - placebo vs. 5:WT - lithium	-0,7146	-1,007 to -0,4226	Yes	****	<0,0001
1:$2b4^{he}2b5^{ho}$ - placebo vs. 5:$2b4^{he}2b5^{ho}$ - lithium	-0,1625	-0,4544 to 0,1295	No	ns	0,9249
1:$2b4^{he}2b5^{ho}$ - placebo vs. 6:WT - placebo	0,05927	-0,2327 to 0,3512	No	ns	>0,9999
1:$2b4^{he}2b5^{ho}$ - placebo vs. 6:$2b4^{he}2b5^{ho}$ - placebo	0,04938	-0,1707 to 0,2694	No	ns	>0,9999
1:$2b4^{he}2b5^{ho}$ - placebo vs. 6:WT - lithium	-1,02	-1,312 to -0,7277	Yes	****	<0,0001
1:$2b4^{he}2b5^{ho}$ - placebo vs. 6:$2b4^{he}2b5^{ho}$ - lithium	-0,2557	-0,5477 to 0,03629	No	ns	0,1764
1:$2b4^{he}2b5^{ho}$ - placebo vs. 7:WT - placebo	0,02248	-0,2695 to 0,3145	No	ns	>0,9999
1:$2b4^{he}2b5^{ho}$ - placebo vs. 7:$2b4^{he}2b5^{ho}$ - placebo	0,05306	-0,1670 to 0,2731	No	ns	>0,9999
1:$2b4^{he}2b5^{ho}$ - placebo vs. 7:WT - lithium	-1,227	-1,519 to -0,9348	Yes	****	<0,0001
1:$2b4^{he}2b5^{ho}$ - placebo vs. 7:$2b4^{he}2b5^{ho}$ - lithium	-0,499	-0,8061 to -0,1919	Yes	****	<0,0001
1:WT - lithium vs. 1:$2b4^{he}2b5^{ho}$ - lithium	0,03876	-0,2684 to 0,3459	No	ns	>0,9999
1:WT - lithium vs. 2:WT - placebo	0,01179	-0,2802 to 0,3038	No	ns	>0,9999
1:WT - lithium vs. 2:$2b4^{he}2b5^{ho}$ - placebo	0,01221	-0,2798 to 0,3042	No	ns	>0,9999
1:WT - lithium vs. 2:WT - lithium	-0,06123	-0,2813 to 0,1588	No	ns	>0,9999
1:WT - lithium vs. 2:$2b4^{he}2b5^{ho}$ - lithium	0,03832	-0,2537 to 0,3303	No	ns	>0,9999
1:WT - lithium vs. 3:WT - placebo	0,04273	-0,2492 to 0,3347	No	ns	>0,9999
1:WT - lithium vs. 3:$2b4^{he}2b5^{ho}$ - placebo	0,05049	-0,2415 to 0,3425	No	ns	>0,9999
1:WT - lithium vs. 3:WT - lithium	-0,1257	-0,3457 to 0,09435	No	ns	0,8967
1:WT - lithium vs. 3:$2b4^{he}2b5^{ho}$ - lithium	0,01961	-0,2724 to 0,3116	No	ns	>0,9999
1:WT - lithium vs. 4:WT - placebo	0,03301	-0,2590 to 0,3250	No	ns	>0,9999
1:WT - lithium vs. 4:$2b4^{he}2b5^{ho}$ - placebo	0,02819	-0,2638 to 0,3202	No	ns	>0,9999
1:WT - lithium vs. 4:WT - lithium	-0,3561	-0,5761 to -0,1361	Yes	****	<0,0001
1:WT - lithium vs. 4:$2b4^{he}2b5^{ho}$ - lithium	-0,06975	-0,3617 to 0,2222	No	ns	>0,9999
1:WT - lithium vs. 5:WT - placebo	-0,01841	-0,3104 to 0,2736	No	ns	>0,9999
1:WT - lithium vs. 5:$2b4^{he}2b5^{ho}$ - placebo	0,02242	-0,2696 to 0,3144	No	ns	>0,9999
1:WT - lithium vs. 5:WT - lithium	-0,7467	-0,9667 to -0,5266	Yes	****	<0,0001
1:WT - lithium vs. 5:$2b4^{he}2b5^{ho}$ - lithium	-0,1946	-0,4865 to 0,09741	No	ns	0,696
1:WT - lithium vs. 6:WT - placebo	0,02716	-0,2648 to 0,3191	No	ns	>0,9999
1:WT - lithium vs. 6:$2b4^{he}2b5^{ho}$ - placebo	0,01728	-0,2747 to 0,3092	No	ns	>0,9999
1:WT - lithium vs. 6:WT - lithium	-1,052	-1,272 to -0,8318	Yes	****	<0,0001
1:WT - lithium vs. 6:$2b4^{he}2b5^{ho}$ - lithium	-0,2878	-0,5798 to 0,004192	No	ns	0,0586
1:WT - lithium vs. 7:WT - placebo	-0,00962	-0,3016 to 0,2824	No	ns	>0,9999
1:WT - lithium vs. 7:$2b4^{he}2b5^{ho}$ - placebo	0,02096	-0,2710 to 0,3129	No	ns	>0,9999
1:WT - lithium vs. 7:WT - lithium	-1,259	-1,479 to -1,039	Yes	****	<0,0001
1:WT - lithium vs. 7:$2b4^{he}2b5^{ho}$ - lithium	-0,5311	-0,8382 to -0,2240	Yes	****	<0,0001
1:$2b4^{he}2b5^{ho}$ - lithium vs. 2:WT - placebo	-0,02697	-0,3341 to 0,2802	No	ns	>0,9999
1:$2b4^{he}2b5^{ho}$ - lithium vs. 2:$2b4^{he}2b5^{ho}$ - placebo	-0,02655	-0,3337 to 0,2806	No	ns	>0,9999
1:$2b4^{he}2b5^{ho}$ - lithium vs. 2:WT - lithium	-0,09999	-0,4071 to 0,2071	No	ns	>0,9999
1:$2b4^{he}2b5^{ho}$ - lithium vs. 2:$2b4^{he}2b5^{ho}$ - lithium	-0,0004402	-0,2405 to 0,2396	No	ns	>0,9999
1:$2b4^{he}2b5^{ho}$ - lithium vs. 3:WT - placebo	0,003967	-0,3032 to 0,3111	No	ns	>0,9999
1:$2b4^{he}2b5^{ho}$ - lithium vs. 3:$2b4^{he}2b5^{ho}$ - placebo	0,01173	-0,2954 to 0,3189	No	ns	>0,9999
1:$2b4^{he}2b5^{ho}$ - lithium vs. 3:WT - lithium	-0,1644	-0,4716 to 0,1427	No	ns	0,9488
1:$2b4^{he}2b5^{ho}$ - lithium vs. 3:$2b4^{he}2b5^{ho}$ - lithium	-0,01915	-0,2592 to 0,2209	No	ns	>0,9999
1:$2b4^{he}2b5^{ho}$ - lithium vs. 4:WT - placebo	-0,005755	-0,3129 to 0,3014	No	ns	>0,9999
1:$2b4^{he}2b5^{ho}$ - lithium vs. 4:$2b4^{he}2b5^{ho}$ - placebo	-0,01057	-0,3177 to 0,2966	No	ns	>0,9999
1:$2b4^{he}2b5^{ho}$ - lithium vs. 4:WT - lithium	-0,3949	-0,7020 to -0,08774	Yes	**	0,0012
1:$2b4^{he}2b5^{ho}$ - lithium vs. 4:$2b4^{he}2b5^{ho}$ - lithium	-0,1085	-0,3485 to 0,1315	No	ns	0,9922
1:$2b4^{he}2b5^{ho}$ - lithium vs. 5:WT - placebo	-0,05717	-0,3643 to 0,2500	No	ns	>0,9999
1:$2b4^{he}2b5^{ho}$ - lithium vs. 5:$2b4^{he}2b5^{ho}$ - placebo	-0,01634	-0,3235 to 0,2908	No	ns	>0,9999
1:$2b4^{he}2b5^{ho}$ - lithium vs. 5:WT - lithium	-0,7854	-1,093 to -0,4783	Yes	****	<0,0001
1:$2b4^{he}2b5^{ho}$ - lithium vs. 5:$2b4^{he}2b5^{ho}$ - lithium	-0,2333	-0,4733 to 0,006699	No	ns	0,0676
1:$2b4^{he}2b5^{ho}$ - lithium vs. 6:WT - placebo	-0,0116	-0,3187 to 0,2955	No	ns	>0,9999
1:$2b4^{he}2b5^{ho}$ - lithium vs. 6:$2b4^{he}2b5^{ho}$ - placebo	-0,02148	-0,3286 to 0,2857	No	ns	>0,9999
1:$2b4^{he}2b5^{ho}$ - lithium vs. 6:WT - lithium	-1,091	-1,398 to -0,7834	Yes	****	<0,0001
1:$2b4^{he}2b5^{ho}$ - lithium vs. 6:$2b4^{he}2b5^{ho}$ - lithium	-0,3265	-0,5666 to -0,08652	Yes	***	0,0005
1:$2b4^{he}2b5^{ho}$ - lithium vs. 7:WT - placebo	-0,04838	-0,3555 to 0,2588	No	ns	>0,9999
1:$2b4^{he}2b5^{ho}$ - lithium vs. 7:$2b4^{he}2b5^{ho}$ - placebo	-0,0178	-0,3249 to 0,2893	No	ns	>0,9999
1:$2b4^{he}2b5^{ho}$ - lithium vs. 7:WT - lithium	-1,298	-1,605 to -0,9905	Yes	****	<0,0001
1:$2b4^{he}2b5^{ho}$ - lithium vs. 7:$2b4^{he}2b5^{ho}$ - lithium	-0,5699	-0,8300 to -0,3098	Yes	****	<0,0001

2:WT - placebo vs. 2:$2b4^{he}$ $2b5^{ho}$ - placebo	0,0004225	-0,2916 to 0,2924	No	ns	>0,9999
2:WT - placebo vs. 2:WT - lithium	-0,07302	-0,3650 to 0,2190	No	ns	>0,9999
2:WT - placebo vs. 2:$2b4^{he}$ $2b5^{ho}$ - lithium	0,02653	-0,2654 to 0,3185	No	ns	>0,9999
2:WT - placebo vs. 3:WT - placebo	0,03094	-0,1891 to 0,2510	No	ns	>0,9999
2:WT - placebo vs. 3:$2b4^{he}$ $2b5^{ho}$ - placebo	0,03871	-0,2533 to 0,3307	No	ns	>0,9999
2:WT - placebo vs. 3:WT - lithium	-0,1375	-0,4294 to 0,1545	No	ns	0,9884
2:WT - placebo vs. 3:$2b4^{he}$ $2b5^{ho}$ - lithium	0,007826	-0,2841 to 0,2998	No	ns	>0,9999
2:WT - placebo vs. 4:WT - placebo	0,02122	-0,1988 to 0,2412	No	ns	>0,9999
2:WT - placebo vs. 4:$2b4^{he}$ $2b5^{ho}$ - placebo	0,01641	-0,2756 to 0,3084	No	ns	>0,9999
2:WT - placebo vs. 4:WT - lithium	-0,3679	-0,6599 to -0,07593	Yes	**	0,0018
2:WT - placebo vs. 4:$2b4^{he}$ $2b5^{ho}$ - lithium	-0,08153	-0,3735 to 0,2104	No	ns	>0,9999
2:WT - placebo vs. 5:WT - placebo	-0,0302	-0,2502 to 0,1898	No	ns	>0,9999
2:WT - placebo vs. 5:$2b4^{he}$ $2b5^{ho}$ - placebo	0,01063	-0,2813 to 0,3026	No	ns	>0,9999
2:WT - placebo vs. 5:WT - lithium	-0,7585	-1,050 to -0,4665	Yes	****	<0,0001
2:WT - placebo vs. 5:$2b4^{he}$ $2b5^{ho}$ - lithium	-0,2064	-0,4983 to 0,08562	No	ns	0,5816
2:WT - placebo vs. 6:WT - placebo	0,01538	-0,2047 to 0,2354	No	ns	>0,9999
2:WT - placebo vs. 6:$2b4^{he}$ $2b5^{ho}$ - placebo	0,005488	-0,2865 to 0,2975	No	ns	>0,9999
2:WT - placebo vs. 6:WT - lithium	-1,064	-1,356 to -0,7716	Yes	****	<0,0001
2:WT - placebo vs. 6:$2b4^{he}$ $2b5^{ho}$ - lithium	-0,2996	-0,5915 to -0,007597	Yes	*	0,0372
2:WT - placebo vs. 7:WT - placebo	-0,02141	-0,2414 to 0,1986	No	ns	>0,9999
2:WT - placebo vs. 7:$2b4^{he}$ $2b5^{ho}$ - placebo	0,009173	-0,2828 to 0,3011	No	ns	>0,9999
2:WT - placebo vs. 7:WT - lithium	-1,271	-1,563 to -0,9787	Yes	****	<0,0001
2:WT - placebo vs. 7:$2b4^{he}$ $2b5^{ho}$ - lithium	-0,5429	-0,8500 to -0,2358	Yes	****	<0,0001
2:$2b4^{he}$ $2b5^{ho}$ - placebo vs. 2:WT - lithium	-0,07344	-0,3654 to 0,2185	No	ns	>0,9999
2:$2b4^{he}$ $2b5^{ho}$ - placebo vs. 2:$2b4^{he}$ $2b5^{ho}$ - lithium	0,02611	-0,2659 to 0,3181	No	ns	>0,9999
2:$2b4^{he}$ $2b5^{ho}$ - placebo vs. 3:WT - placebo	0,03052	-0,2615 to 0,3225	No	ns	>0,9999
2:$2b4^{he}$ $2b5^{ho}$ - placebo vs. 3:$2b4^{he}$ $2b5^{ho}$ - placebo	0,03828	-0,1817 to 0,2583	No	ns	>0,9999
2:$2b4^{he}$ $2b5^{ho}$ - placebo vs. 3:WT - lithium	-0,1379	-0,4299 to 0,1541	No	ns	0,988
2:$2b4^{he}$ $2b5^{ho}$ - placebo vs. 3:$2b4^{he}$ $2b5^{ho}$ - lithium	0,007403	-0,2846 to 0,2994	No	ns	>0,9999
2:$2b4^{he}$ $2b5^{ho}$ - placebo vs. 4:WT - placebo	0,0208	-0,2712 to 0,3128	No	ns	>0,9999
2:$2b4^{he}$ $2b5^{ho}$ - placebo vs. 4:$2b4^{he}$ $2b5^{ho}$ - placebo	0,01598	-0,2040 to 0,2360	No	ns	>0,9999
2:$2b4^{he}$ $2b5^{ho}$ - placebo vs. 4:WT - lithium	-0,3683	-0,6603 to -0,07635	Yes	**	0,0017
2:$2b4^{he}$ $2b5^{ho}$ - placebo vs. 4:$2b4^{he}$ $2b5^{ho}$ - lithium	-0,08196	-0,3739 to 0,2100	No	ns	>0,9999
2:$2b4^{he}$ $2b5^{ho}$ - placebo vs. 5:WT - placebo	-0,03062	-0,3226 to 0,2613	No	ns	>0,9999
2:$2b4^{he}$ $2b5^{ho}$ - placebo vs. 5:$2b4^{he}$ $2b5^{ho}$ - placebo	0,01021	-0,2098 to 0,2302	No	ns	>0,9999
2:$2b4^{he}$ $2b5^{ho}$ - placebo vs. 5:WT - lithium	-0,7589	-1,051 to -0,4669	Yes	****	<0,0001
2:$2b4^{he}$ $2b5^{ho}$ - placebo vs. 5:$2b4^{he}$ $2b5^{ho}$ - lithium	-0,2068	-0,4987 to 0,08520	No	ns	0,5774
2:$2b4^{he}$ $2b5^{ho}$ - placebo vs. 6:WT - placebo	0,01495	-0,2770 to 0,3069	No	ns	>0,9999
2:$2b4^{he}$ $2b5^{ho}$ - placebo vs. 6:$2b4^{he}$ $2b5^{ho}$ - placebo	0,005065	-0,2150 to 0,2251	No	ns	>0,9999
2:$2b4^{he}$ $2b5^{ho}$ - placebo vs. 6:WT - lithium	-1,064	-1,356 to -0,7720	Yes	****	<0,0001
2:$2b4^{he}$ $2b5^{ho}$ - placebo vs. 6:$2b4^{he}$ $2b5^{ho}$ - lithium	-0,3	-0,5920 to -0,008019	Yes	*	0,0365
2:$2b4^{he}$ $2b5^{ho}$ - placebo vs. 7:WT - placebo	-0,02183	-0,3138 to 0,2701	No	ns	>0,9999
2:$2b4^{he}$ $2b5^{ho}$ - placebo vs. 7:$2b4^{he}$ $2b5^{ho}$ - placebo	0,00875	-0,2113 to 0,2288	No	ns	>0,9999
2:$2b4^{he}$ $2b5^{ho}$ - placebo vs. 7:WT - lithium	-1,271	-1,563 to -0,9791	Yes	****	<0,0001
2:$2b4^{he}$ $2b5^{ho}$ - placebo vs. 7:$2b4^{he}$ $2b5^{ho}$ - lithium	-0,5433	-0,8505 to -0,2362	Yes	****	<0,0001
2:WT - lithium vs. 2:$2b4^{he}$ $2b5^{ho}$ - lithium	0,09955	-0,1924 to 0,3915	No	ns	>0,9999
2:WT - lithium vs. 3:WT - placebo	0,104	-0,1880 to 0,3959	No	ns	0,9998
2:WT - lithium vs. 3:$2b4^{he}$ $2b5^{ho}$ - placebo	0,1117	-0,1802 to 0,4037	No	ns	0,9994
2:WT - lithium vs. 3:WT - lithium	-0,06445	-0,2845 to 0,1556	No	ns	>0,9999
2:WT - lithium vs. 3:$2b4^{he}$ $2b5^{ho}$ - lithium	0,08085	-0,2111 to 0,3728	No	ns	>0,9999
2:WT - lithium vs. 4:WT - placebo	0,09424	-0,1977 to 0,3862	No	ns	>0,9999
2:WT - lithium vs. 4:$2b4^{he}$ $2b5^{ho}$ - placebo	0,08943	-0,2025 to 0,3814	No	ns	>0,9999
2:WT - lithium vs. 4:WT - lithium	-0,2949	-0,5149 to -0,07485	Yes	***	0,0006
2:WT - lithium vs. 4:$2b4^{he}$ $2b5^{ho}$ - lithium	-0,008511	-0,3005 to 0,2835	No	ns	>0,9999
2:WT - lithium vs. 5:WT - placebo	0,04282	-0,2492 to 0,3348	No	ns	>0,9999
2:WT - lithium vs. 5:$2b4^{he}$ $2b5^{ho}$ - placebo	0,08365	-0,2083 to 0,3756	No	ns	>0,9999
2:WT - lithium vs. 5:WT - lithium	-0,6854	-0,9055 to -0,4654	Yes	****	<0,0001
2:WT - lithium vs. 5:$2b4^{he}$ $2b5^{ho}$ - lithium	-0,1333	-0,4253 to 0,1586	No	ns	0,9923
2:WT - lithium vs. 6:WT - placebo	0,0884	-0,2036 to 0,3804	No	ns	>0,9999
2:WT - lithium vs. 6:$2b4^{he}$ $2b5^{ho}$ - placebo	0,07851	-0,2135 to 0,3705	No	ns	>0,9999
2:WT - lithium vs. 6:WT - lithium	-0,9906	-1,211 to -0,7705	Yes	****	<0,0001
2:WT - lithium vs. 6:$2b4^{he}$ $2b5^{ho}$ - lithium	-0,2265	-0,5185 to 0,06543	No	ns	0,3876
2:WT - lithium vs. 7:WT - placebo	0,05161	-0,2404 to 0,3436	No	ns	>0,9999
2:WT - lithium vs. 7:$2b4^{he}$ $2b5^{ho}$ - placebo	0,08219	-0,2098 to 0,3742	No	ns	>0,9999
2:WT - lithium vs. 7:WT - lithium	-1,198	-1,418 to -0,9776	Yes	****	<0,0001
2:WT - lithium vs. 7:$2b4^{he}$ $2b5^{ho}$ - lithium	-0,4699	-0,7770 to -0,1627	Yes	****	<0,0001
2:$2b4^{he}$ $2b5^{ho}$ - lithium vs. 3:WT - placebo	0,004407	-0,2876 to 0,2964	No	ns	>0,9999
2:$2b4^{he}$ $2b5^{ho}$ - lithium vs. 3:$2b4^{he}$ $2b5^{ho}$ - placebo	0,01217	-0,2798 to 0,3041	No	ns	>0,9999
2:$2b4^{he}$ $2b5^{ho}$ - lithium vs. 3:WT - lithium	-0,164	-0,4560 to 0,1280	No	ns	0,9178
2:$2b4^{he}$ $2b5^{ho}$ - lithium vs. 3:$2b4^{he}$ $2b5^{ho}$ - lithium	-0,01871	-0,2387 to 0,2013	No	ns	>0,9999
2:$2b4^{he}$ $2b5^{ho}$ - lithium vs. 4:WT - placebo	-0,005315	-0,2973 to 0,2867	No	ns	>0,9999
2:$2b4^{he}$ $2b5^{ho}$ - lithium vs. 4:$2b4^{he}$ $2b5^{ho}$ - placebo	-0,01013	-0,3021 to 0,2818	No	ns	>0,9999
2:$2b4^{he}$ $2b5^{ho}$ - lithium vs. 4:WT - lithium	-0,3944	-0,6864 to -0,1025	Yes	***	0,0005
2:$2b4^{he}$ $2b5^{ho}$ - lithium vs. 4:$2b4^{he}$ $2b5^{ho}$ - lithium	-0,1081	-0,3281 to 0,1120	No	ns	0,978
2:$2b4^{he}$ $2b5^{ho}$ - lithium vs. 5:WT - placebo	-0,05673	-0,3487 to 0,2352	No	ns	>0,9999
2:$2b4^{he}$ $2b5^{ho}$ - lithium vs. 5:$2b4^{he}$ $2b5^{ho}$ - placebo	-0,0159	-0,3079 to 0,2761	No	ns	>0,9999
2:$2b4^{he}$ $2b5^{ho}$ - lithium vs. 5:WT - lithium	-0,785	-1,077 to -0,4930	Yes	****	<0,0001
2:$2b4^{he}$ $2b5^{ho}$ - lithium vs. 5:$2b4^{he}$ $2b5^{ho}$ - lithium	-0,2329	-0,4529 to -0,01285	Yes	*	0,0257
2:$2b4^{he}$ $2b5^{ho}$ - lithium vs. 6:WT - placebo	-0,01116	-0,3031 to 0,2808	No	ns	>0,9999
2:$2b4^{he}$ $2b5^{ho}$ - lithium vs. 6:$2b4^{he}$ $2b5^{ho}$ - placebo	-0,02104	-0,3130 to 0,2709	No	ns	>0,9999
2:$2b4^{he}$ $2b5^{ho}$ - lithium vs. 6:WT - lithium	-1,09	-1,382 to -0,7981	Yes	****	<0,0001
2:$2b4^{he}$ $2b5^{ho}$ - lithium vs. 6:$2b4^{he}$ $2b5^{ho}$ - lithium	-0,3261	-0,5461 to -0,1061	Yes	****	<0,0001
2:$2b4^{he}$ $2b5^{ho}$ - lithium vs. 7:WT - placebo	-0,04794	-0,3399 to 0,2440	No	ns	>0,9999
2:$2b4^{he}$ $2b5^{ho}$ - lithium vs. 7:$2b4^{he}$ $2b5^{ho}$ - placebo	-0,01736	-0,3093 to 0,2746	No	ns	>0,9999
2:$2b4^{he}$ $2b5^{ho}$ - lithium vs. 7:WT - lithium	-1,297	-1,589 to -1,005	Yes	****	<0,0001
2:$2b4^{he}$ $2b5^{ho}$ - lithium vs. 7:$2b4^{he}$ $2b5^{ho}$ - lithium	-0,5694	-0,8094 to -0,3294	Yes	****	<0,0001

3:WT - placebo vs. 3:$2b4^{he} 2b5^{ho}$ - placebo	0,007767	-0,2842 to 0,2997	No	ns	>0,9999	
3:WT - placebo vs. 3:WT - lithium	-0,1684	-0,4604 to 0,1236	No	ns		0,8954
3:WT - placebo vs. 3:$2b4^{he} 2b5^{ho}$ - lithium	-0,02311	-0,3151 to 0,2689	No	ns	>0,9999	
3:WT - placebo vs. 4:WT - placebo	-0,009722	-0,2298 to 0,2103	No	ns	>0,9999	
3:WT - placebo vs. 4:$2b4^{he} 2b5^{ho}$ - placebo	-0,01453	-0,3065 to 0,2774	No	ns	>0,9999	
3:WT - placebo vs. 4:WT - lithium	-0,3988	-0,6908 to -0,1069	Yes	***		0,0004
3:WT - placebo vs. 4:$2b4^{he} 2b5^{ho}$ - lithium	-0,1125	-0,4044 to 0,1795	No	ns		0,9994
3:WT - placebo vs. 5:WT - placebo	-0,06114	-0,2812 to 0,1589	No	ns	>0,9999	
3:WT - placebo vs. 5:$2b4^{he} 2b5^{ho}$ - placebo	-0,02031	-0,3123 to 0,2717	No	ns	>0,9999	
3:WT - placebo vs. 5:WT - lithium	-0,7894	-1,081 to -0,4974	Yes	****	<0,0001	
3:WT - placebo vs. 5:$2b4^{he} 2b5^{ho}$ - lithium	-0,2373	-0,5293 to 0,05468	No	ns		0,2977
3:WT - placebo vs. 6:WT - placebo	-0,01556	-0,2356 to 0,2045	No	ns	>0,9999	
3:WT - placebo vs. 6:$2b4^{he} 2b5^{ho}$ - placebo	-0,02545	-0,3174 to 0,2665	No	ns	>0,9999	
3:WT - placebo vs. 6:WT - lithium	-1,095	-1,386 to -0,8025	Yes	****	<0,0001	
3:WT - placebo vs. 6:$2b4^{he} 2b5^{ho}$ - lithium	-0,3305	-0,6225 to -0,03854	Yes	*		0,0101
3:WT - placebo vs. 7:WT - placebo	-0,05235	-0,2724 to 0,1677	No	ns	>0,9999	
3:WT - placebo vs. 7:$2b4^{he} 2b5^{ho}$ - placebo	-0,02177	-0,3137 to 0,2702	No	ns	>0,9999	
3:WT - placebo vs. 7:WT - lithium	-1,302	-1,594 to -1,010	Yes	****	<0,0001	
3:WT - placebo vs. 7:$2b4^{he} 2b5^{ho}$ - lithium	-0,5738	-0,8810 to -0,2667	Yes	****	<0,0001	
3:$2b4^{he} 2b5^{ho}$ - placebo vs. 3:WT - lithium	-0,1762	-0,4682 to 0,1158	No	ns		0,8474
3:$2b4^{he} 2b5^{ho}$ - placebo vs. 3:$2b4^{he} 2b5^{ho}$ - lithium	-0,03088	-0,3229 to 0,2611	No	ns	>0,9999	
3:$2b4^{he} 2b5^{ho}$ - placebo vs. 4:WT - placebo	-0,01749	-0,3095 to 0,2745	No	ns	>0,9999	
3:$2b4^{he} 2b5^{ho}$ - placebo vs. 4:$2b4^{he} 2b5^{ho}$ - placebo	-0,0223	-0,2423 to 0,1977	No	ns	>0,9999	
3:$2b4^{he} 2b5^{ho}$ - placebo vs. 4:WT - lithium	-0,4066	-0,6986 to -0,1146	Yes	***		0,0002
3:$2b4^{he} 2b5^{ho}$ - placebo vs. 4:$2b4^{he} 2b5^{ho}$ - lithium	-0,1202	-0,4122 to 0,1717	No	ns		0,9982
3:$2b4^{he} 2b5^{ho}$ - placebo vs. 5:WT - placebo	-0,06891	-0,3609 to 0,2231	No	ns	>0,9999	
3:$2b4^{he} 2b5^{ho}$ - placebo vs. 5:$2b4^{he} 2b5^{ho}$ - placebo	-0,02808	-0,2481 to 0,1920	No	ns	>0,9999	
3:$2b4^{he} 2b5^{ho}$ - placebo vs. 5:WT - lithium	-0,7972	-1,089 to -0,5052	Yes	****	<0,0001	
3:$2b4^{he} 2b5^{ho}$ - placebo vs. 5:$2b4^{he} 2b5^{ho}$ - lithium	-0,2451	-0,5370 to 0,04692	No	ns		0,2412
3:$2b4^{he} 2b5^{ho}$ - placebo vs. 6:WT - placebo	-0,02333	-0,3153 to 0,2686	No	ns	>0,9999	
3:$2b4^{he} 2b5^{ho}$ - placebo vs. 6:$2b4^{he} 2b5^{ho}$ - placebo	-0,03322	-0,2532 to 0,1868	No	ns	>0,9999	
3:$2b4^{he} 2b5^{ho}$ - placebo vs. 6:WT - lithium	-1,102	-1,394 to -0,8103	Yes	****	<0,0001	
3:$2b4^{he} 2b5^{ho}$ - placebo vs. 6:$2b4^{he} 2b5^{ho}$ - lithium	-0,3383	-0,6303 to -0,04630	Yes	**		0,0071
3:$2b4^{he} 2b5^{ho}$ - placebo vs. 7:WT - placebo	-0,06011	-0,3521 to 0,2319	No	ns	>0,9999	
3:$2b4^{he} 2b5^{ho}$ - placebo vs. 7:$2b4^{he} 2b5^{ho}$ - placebo	-0,02953	-0,2496 to 0,1905	No	ns	>0,9999	
3:$2b4^{he} 2b5^{ho}$ - placebo vs. 7:WT - lithium	-1,309	-1,601 to -1,017	Yes	****	<0,0001	
3:$2b4^{he} 2b5^{ho}$ - placebo vs. 7:$2b4^{he} 2b5^{ho}$ - lithium	-0,5816	-0,8887 to -0,2745	Yes	****	<0,0001	
3:WT - lithium vs. 3:$2b4^{he} 2b5^{ho}$ - lithium	0,1453	-0,1467 to 0,4373	No	ns		0,9772
3:WT - lithium vs. 4:WT - placebo	0,1587	-0,1333 to 0,4507	No	ns		0,9403
3:WT - lithium vs. 4:$2b4^{he} 2b5^{ho}$ - placebo	0,1539	-0,1381 to 0,4459	No	ns		0,9566
3:WT - lithium vs. 4:WT - lithium	-0,2304	-0,4505 to -0,01040	Yes	*		0,0293
3:WT - lithium vs. 4:$2b4^{he} 2b5^{ho}$ - lithium	0,05594	-0,2360 to 0,3479	No	ns	>0,9999	
3:WT - lithium vs. 5:WT - placebo	0,1073	-0,1847 to 0,3992	No	ns		0,9997
3:WT - lithium vs. 5:$2b4^{he} 2b5^{ho}$ - placebo	0,1481	-0,1439 to 0,4401	No	ns		0,9715
3:WT - lithium vs. 5:WT - lithium	-0,621	-0,8410 to -0,4010	Yes	****	<0,0001	
3:WT - lithium vs. 5:$2b4^{he} 2b5^{ho}$ - lithium	-0,06888	-0,3609 to 0,2231	No	ns	>0,9999	
3:WT - lithium vs. 6:WT - placebo	0,1528	-0,1391 to 0,4448	No	ns		0,9596
3:WT - lithium vs. 6:$2b4^{he} 2b5^{ho}$ - placebo	0,143	-0,1490 to 0,4349	No	ns		0,9812
3:WT - lithium vs. 6:WT - lithium	-0,9261	-1,146 to -0,7061	Yes	****	<0,0001	
3:WT - lithium vs. 6:$2b4^{he} 2b5^{ho}$ - lithium	-0,1621	-0,4541 to 0,1299	No	ns		0,9265
3:WT - lithium vs. 7:WT - placebo	0,1161	-0,1759 to 0,4080	No	ns		0,999
3:WT - lithium vs. 7:$2b4^{he} 2b5^{ho}$ - placebo	0,1466	-0,1453 to 0,4386	No	ns		0,9746
3:WT - lithium vs. 7:WT - lithium	-1,133	-1,353 to -0,9132	Yes	****	<0,0001	
3:WT - lithium vs. 7:$2b4^{he} 2b5^{ho}$ - lithium	-0,4054	-0,7126 to -0,09828	Yes	***		0,0007
3:$2b4^{he} 2b5^{ho}$ - lithium vs. 4:WT - placebo	0,01339	-0,2786 to 0,3054	No	ns	>0,9999	
3:$2b4^{he} 2b5^{ho}$ - lithium vs. 4:$2b4^{he} 2b5^{ho}$ - placebo	0,008581	-0,2834 to 0,3006	No	ns	>0,9999	
3:$2b4^{he} 2b5^{ho}$ - lithium vs. 4:WT - lithium	-0,3757	-0,6677 to -0,08376	Yes	**		0,0012
3:$2b4^{he} 2b5^{ho}$ - lithium vs. 4:$2b4^{he} 2b5^{ho}$ - lithium	-0,08936	-0,3094 to 0,1307	No	ns		0,9983
3:$2b4^{he} 2b5^{ho}$ - lithium vs. 5:WT - placebo	-0,03803	-0,3300 to 0,2539	No	ns	>0,9999	
3:$2b4^{he} 2b5^{ho}$ - lithium vs. 5:$2b4^{he} 2b5^{ho}$ - placebo	0,002803	-0,2892 to 0,2948	No	ns	>0,9999	
3:$2b4^{he} 2b5^{ho}$ - lithium vs. 5:WT - lithium	-0,7663	-1,058 to -0,4743	Yes	****	<0,0001	
3:$2b4^{he} 2b5^{ho}$ - lithium vs. 5:$2b4^{he} 2b5^{ho}$ - lithium	-0,2142	-0,4342 to 0,005855	No	ns		0,0666
3:$2b4^{he} 2b5^{ho}$ - lithium vs. 6:WT - placebo	0,00755	-0,2844 to 0,2995	No	ns	>0,9999	
3:$2b4^{he} 2b5^{ho}$ - lithium vs. 6:$2b4^{he} 2b5^{ho}$ - placebo	-0,002338	-0,2943 to 0,2896	No	ns	>0,9999	
3:$2b4^{he} 2b5^{ho}$ - lithium vs. 6:WT - lithium	-1,071	-1,363 to -0,7794	Yes	****	<0,0001	
3:$2b4^{he} 2b5^{ho}$ - lithium vs. 6:$2b4^{he} 2b5^{ho}$ - lithium	-0,3074	-0,5274 to -0,08736	Yes	***		0,0003
3:$2b4^{he} 2b5^{ho}$ - lithium vs. 7:WT - placebo	-0,02923	-0,3212 to 0,2627	No	ns	>0,9999	
3:$2b4^{he} 2b5^{ho}$ - lithium vs. 7:$2b4^{he} 2b5^{ho}$ - placebo	0,001347	-0,2906 to 0,2933	No	ns	>0,9999	
3:$2b4^{he} 2b5^{ho}$ - lithium vs. 7:WT - lithium	-1,279	-1,570 to -0,9865	Yes	****	<0,0001	
3:$2b4^{he} 2b5^{ho}$ - lithium vs. 7:$2b4^{he} 2b5^{ho}$ - lithium	-0,5507	-0,7907 to -0,3107	Yes	****	<0,0001	
4:WT - placebo vs. 4:$2b4^{he} 2b5^{ho}$ - placebo	-0,004811	-0,2968 to 0,2872	No	ns	>0,9999	
4:WT - placebo vs. 4:WT - lithium	-0,3891	-0,6811 to -0,09715	Yes	***		0,0006
4:WT - placebo vs. 4:$2b4^{he} 2b5^{ho}$ - lithium	-0,1028	-0,3947 to 0,1892	No	ns		0,9999
4:WT - placebo vs. 5:WT - placebo	-0,05142	-0,2714 to 0,1686	No	ns	>0,9999	
4:WT - placebo vs. 5:$2b4^{he} 2b5^{ho}$ - placebo	-0,01059	-0,3026 to 0,2814	No	ns	>0,9999	
4:WT - placebo vs. 5:WT - lithium	-0,7797	-1,072 to -0,4877	Yes	****	<0,0001	
4:WT - placebo vs. 5:$2b4^{he} 2b5^{ho}$ - lithium	-0,2276	-0,5195 to 0,06441	No	ns		0,3786
4:WT - placebo vs. 6:WT - placebo	-0,005842	-0,2259 to 0,2142	No	ns	>0,9999	
4:WT - placebo vs. 6:$2b4^{he} 2b5^{ho}$ - placebo	-0,01573	-0,3077 to 0,2762	No	ns	>0,9999	
4:WT - placebo vs. 6:WT - lithium	-1,085	-1,377 to -0,7928	Yes	****	<0,0001	
4:WT - placebo vs. 6:$2b4^{he} 2b5^{ho}$ - lithium	-0,3208	-0,6128 to -0,02881	Yes	*		0,0154
4:WT - placebo vs. 7:WT - placebo	-0,04263	-0,2627 to 0,1774	No	ns	>0,9999	
4:WT - placebo vs. 7:$2b4^{he} 2b5^{ho}$ - placebo	-0,01205	-0,3040 to 0,2799	No	ns	>0,9999	
4:WT - placebo vs. 7:WT - lithium	-1,292	-1,584 to -0,9999	Yes	****	<0,0001	
4:WT - placebo vs. 7:$2b4^{he} 2b5^{ho}$ - lithium	-0,5641	-0,8713 to -0,2570	Yes	****	<0,0001	
4:$2b4^{he} 2b5^{ho}$ - placebo vs. 4:WT - lithium	-0,3843	-0,6763 to -0,09234	Yes	***		0,0008
4:$2b4^{he} 2b5^{ho}$ - placebo vs. 4:$2b4^{he} 2b5^{ho}$ - lithium	-0,09794	-0,3899 to 0,1940	No	ns	>0,9999	
4:$2b4^{he} 2b5^{ho}$ - placebo vs. 5:WT - placebo	-0,04661	-0,3386 to 0,2454	No	ns	>0,9999	
4:$2b4^{he} 2b5^{ho}$ - placebo vs. 5:$2b4^{he} 2b5^{ho}$ - placebo	-0,005777	-0,2258 to 0,2143	No	ns	>0,9999	
4:$2b4^{he} 2b5^{ho}$ - placebo vs. 5:WT - lithium	-0,7749	-1,067 to -0,4829	Yes	****	<0,0001	
4:$2b4^{he} 2b5^{ho}$ - placebo vs. 5:$2b4^{he} 2b5^{ho}$ - lithium	-0,2228	-0,5147 to 0,06922	No	ns		0,4222
4:$2b4^{he} 2b5^{ho}$ - placebo vs. 6:WT - placebo	-0,001031	-0,2930 to 0,2909	No	ns	>0,9999	
4:$2b4^{he} 2b5^{ho}$ - placebo vs. 6:$2b4^{he} 2b5^{ho}$ - placebo	-0,01092	-0,2309 to 0,2091	No	ns	>0,9999	
4:$2b4^{he} 2b5^{ho}$ - placebo vs. 6:WT - lithium	-1,08	-1,372 to -0,7880	Yes	****	<0,0001	
4:$2b4^{he} 2b5^{ho}$ - placebo vs. 6:$2b4^{he} 2b5^{ho}$ - lithium	-0,316	-0,6079 to -0,02400	Yes	*		0,0189
4:$2b4^{he} 2b5^{ho}$ - placebo vs. 7:WT - placebo	-0,03781	-0,3298 to 0,2542	No	ns	>0,9999	
4:$2b4^{he} 2b5^{ho}$ - placebo vs. 7:$2b4^{he} 2b5^{ho}$ - placebo	-0,007234	-0,2273 to 0,2128	No	ns	>0,9999	
4:$2b4^{he} 2b5^{ho}$ - placebo vs. 7:WT - lithium	-1,287	-1,579 to -0,9951	Yes	****	<0,0001	
4:$2b4^{he} 2b5^{ho}$ - placebo vs. 7:$2b4^{he} 2b5^{ho}$ - lithium	-0,5593	-0,8664 to -0,2522	Yes	****	<0,0001	
4:WT - lithium vs. 4:$2b4^{he} 2b5^{ho}$ - lithium	0,2864	-0,005602 to 0,5783	No	ns		0,0618
4:WT - lithium vs. 5:WT - placebo	0,3377	0,04573 to 0,6297	Yes	**		0,0073
4:WT - lithium vs. 5:$2b4^{he} 2b5^{ho}$ - placebo	0,3785	0,08656 to 0,6705	Yes	**		0,001
4:WT - lithium vs. 5:WT - lithium	-0,3906	-0,6106 to -0,1705	Yes	****	<0,0001	
4:WT - lithium vs. 5:$2b4^{he} 2b5^{ho}$ - lithium	0,1616	-0,1304 to 0,4535	No	ns		0,9288
4:WT - lithium vs. 6:WT - placebo	0,3833	0,09131 to 0,6753	Yes	***		0,0008
4:WT - lithium vs. 6:$2b4^{he} 2b5^{ho}$ - placebo	0,3734	0,08142 to 0,6654	Yes	**		0,0013
4:WT - lithium vs. 6:WT - lithium	-0,6957	-0,9157 to -0,4756	Yes	****	<0,0001	
4:WT - lithium vs. 6:$2b4^{he} 2b5^{ho}$ - lithium	0,06833	-0,2236 to 0,3603	No	ns	>0,9999	
4:WT - lithium vs. 7:WT - placebo	0,3465	0,05452 to 0,6385	Yes	**		0,0049
4:WT - lithium vs. 7:$2b4^{he} 2b5^{ho}$ - placebo	0,3771	0,08510 to 0,6691	Yes	**		0,0011
4:WT - lithium vs. 7:WT - lithium	-0,9028	-1,123 to -0,6828	Yes	****	<0,0001	
4:WT - lithium vs. 7:$2b4^{he} 2b5^{ho}$ - lithium	-0,175	-0,4821 to 0,1322	No	ns		0,9064
4:$2b4^{he} 2b5^{ho}$ - lithium vs. 5:WT - placebo	0,05133	-0,2406 to 0,3433	No	ns	>0,9999	
4:$2b4^{he} 2b5^{ho}$ - lithium vs. 5:$2b4^{he} 2b5^{ho}$ - placebo	0,09216	-0,1998 to 0,3841	No	ns	>0,9999	
4:$2b4^{he} 2b5^{ho}$ - lithium vs. 5:WT - lithium	-0,6769	-0,9689 to -0,3850	Yes	****	<0,0001	
4:$2b4^{he} 2b5^{ho}$ - lithium vs. 5:$2b4^{he} 2b5^{ho}$ - lithium	-0,1248	-0,3448 to 0,09521	No	ns		0,9028
4:$2b4^{he} 2b5^{ho}$ - lithium vs. 6:WT - placebo	0,09691	-0,1951 to 0,3889	No	ns	>0,9999	
4:$2b4^{he} 2b5^{ho}$ - lithium vs. 6:$2b4^{he} 2b5^{ho}$ - placebo	0,08702	-0,2050 to 0,3790	No	ns	>0,9999	
4:$2b4^{he} 2b5^{ho}$ - lithium vs. 6:WT - lithium	-0,982	-1,274 to -0,6901	Yes	****	<0,0001	
4:$2b4^{he} 2b5^{ho}$ - lithium vs. 6:$2b4^{he} 2b5^{ho}$ - lithium	-0,218	-0,4381 to 0,001994	No	ns		0,0552
4:$2b4^{he} 2b5^{ho}$ - lithium vs. 7:WT - placebo	0,06013	-0,2318 to 0,3521	No	ns	>0,9999	
4:$2b4^{he} 2b5^{ho}$ - lithium vs. 7:$2b4^{he} 2b5^{ho}$ - placebo	0,09071	-0,2013 to 0,3827	No	ns	>0,9999	
4:$2b4^{he} 2b5^{ho}$ - lithium vs. 7:WT - lithium	-1,189	-1,481 to -0,8972	Yes	****	<0,0001	
4:$2b4^{he} 2b5^{ho}$ - lithium vs. 7:$2b4^{he} 2b5^{ho}$ - lithium	-0,4614	-0,7014 to -0,2213	Yes	****	<0,0001	
5:WT - placebo vs. 5:$2b4^{he} 2b5^{ho}$ - placebo	0,04083	-0,2511 to 0,3328	No	ns	>0,9999	
5:WT - placebo vs. 5:WT - lithium	-0,7283	-1,020 to -0,4363	Yes	****	<0,0001	
5:WT - placebo vs. 5:$2b4^{he} 2b5^{ho}$ - lithium	-0,1761	-0,4681 to 0,1158	No	ns		0,8476
5:WT - placebo vs. 6:WT - placebo	0,04558	-0,1745 to 0,2656	No	ns	>0,9999	
5:WT - placebo vs. 6:$2b4^{he} 2b5^{ho}$ - placebo	0,03569	-0,2563 to 0,3277	No	ns	>0,9999	
5:WT - placebo vs. 6:WT - lithium	-1,033	-1,325 to -0,7414	Yes	****	<0,0001	
5:WT - placebo vs. 6:$2b4^{he} 2b5^{ho}$ - lithium	-0,2694	-0,5613 to 0,02260	No	ns		0,1133
5:WT - placebo vs. 7:WT - placebo	0,008793	-0,2112 to 0,2288	No	ns	>0,9999	
5:WT - placebo vs. 7:$2b4^{he} 2b5^{ho}$ - placebo	0,03937	-0,2526 to 0,3313	No	ns	>0,9999	
5:WT - placebo vs. 7:WT - lithium	-1,24	-1,532 to -0,9485	Yes	****	<0,0001	
5:WT - placebo vs. 7:$2b4^{he} 2b5^{ho}$ - lithium	-0,5127	-0,8198 to -0,2056	Yes	****	<0,0001	
5:$2b4^{he} 2b5^{ho}$ - placebo vs. 5:WT - lithium	-0,7691	-1,061 to -0,4771	Yes	****	<0,0001	
5:$2b4^{he} 2b5^{ho}$ - placebo vs. 5:$2b4^{he} 2b5^{ho}$ - lithium	-0,217	-0,5090 to 0,07499	No	ns		0,477
5:$2b4^{he} 2b5^{ho}$ - placebo vs. 6:WT - placebo	0,004746	-0,2872 to 0,2967	No	ns	>0,9999	
5:$2b4^{he} 2b5^{ho}$ - placebo vs. 6:$2b4^{he} 2b5^{ho}$ - placebo	-0,005141	-0,2252 to 0,2149	No	ns	>0,9999	
5:$2b4^{he} 2b5^{ho}$ - placebo vs. 6:WT - lithium	-1,074	-1,366 to -0,7822	Yes	****	<0,0001	
5:$2b4^{he} 2b5^{ho}$ - placebo vs. 6:$2b4^{he} 2b5^{ho}$ - lithium	-0,3102	-0,6022 to -0,01823	Yes	*		0,0241
5:$2b4^{he} 2b5^{ho}$ - placebo vs. 7:WT - placebo	-0,03204	-0,3240 to 0,2599	No	ns	>0,9999	
5:$2b4^{he} 2b5^{ho}$ - placebo vs. 7:$2b4^{he} 2b5^{ho}$ - placebo	-0,001457	-0,2215 to 0,2186	No	ns	>0,9999	
5:$2b4^{he} 2b5^{ho}$ - placebo vs. 7:WT - lithium	-1,281	-1,573 to -0,9894	Yes	****	<0,0001	
5:$2b4^{he} 2b5^{ho}$ - placebo vs. 7:$2b4^{he} 2b5^{ho}$ - lithium	-0,5535	-0,8607 to -0,2464	Yes	****	<0,0001	
5:WT - lithium vs. 5:$2b4^{he} 2b5^{ho}$ - lithium	0,5521	0,2601 to 0,8441	Yes	****	<0,0001	
5:WT - lithium vs. 6:WT - placebo	0,7738	0,4819 to 1,066	Yes	****	<0,0001	
5:WT - lithium vs. 6:$2b4^{he} 2b5^{ho}$ - placebo	0,764	0,4720 to 1,056	Yes	****	<0,0001	
5:WT - lithium vs. 6:WT - lithium	-0,3051	-0,5251 to -0,08508	Yes	***		0,0003
5:WT - lithium vs. 6:$2b4^{he} 2b5^{ho}$ - lithium	0,4589	0,1669 to 0,7509	Yes	****	<0,0001	
5:WT - lithium vs. 7:WT - placebo	0,7371	0,4451 to 1,029	Yes	****	<0,0001	
5:WT - lithium vs. 7:$2b4^{he} 2b5^{ho}$ - placebo	0,7676	0,4757 to 1,060	Yes	****	<0,0001	
5:WT - lithium vs. 7:WT - lithium	-0,5122	-0,7323 to -0,2922	Yes	****	<0,0001	
5:WT - lithium vs. 7:$2b4^{he} 2b5^{ho}$ - lithium	0,2156	-0,09157 to 0,5227	No	ns		0,5957
5:$2b4^{he} 2b5^{ho}$ - lithium vs. 6:WT - placebo	0,2217	-0,07025 to 0,5137	No	ns		0,4318
5:$2b4^{he} 2b5^{ho}$ - lithium vs. 6:$2b4^{he} 2b5^{ho}$ - placebo	0,2118	-0,08014 to 0,5038	No	ns		0,5272
5:$2b4^{he} 2b5^{ho}$ - lithium vs. 6:WT - lithium	-0,8572	-1,149 to -0,5652	Yes	****	<0,0001	
5:$2b4^{he} 2b5^{ho}$ - lithium vs. 6:$2b4^{he} 2b5^{ho}$ - lithium	-0,09322	-0,3133 to 0,1268	No	ns		0,9968
5:$2b4^{he} 2b5^{ho}$ - lithium vs. 7:WT - placebo	0,1849	-0,1070 to 0,4769	No	ns		0,781
5:$2b4^{he} 2b5^{ho}$ - lithium vs. 7:$2b4^{he} 2b5^{ho}$ - placebo	0,2155	-0,07645 to 0,5075	No	ns		0,4911
5:$2b4^{he} 2b5^{ho}$ - lithium vs. 7:WT - lithium	-1,064	-1,356 to -0,7724	Yes	****	<0,0001	
5:$2b4^{he} 2b5^{ho}$ - lithium vs. 7:$2b4^{he} 2b5^{ho}$ - lithium	-0,3365	-0,5766 to -0,09652	Yes	***		0,0003
6:WT - placebo vs. 6:$2b4^{he} 2b5^{ho}$ - placebo	-0,009888	-0,3019 to 0,2821	No	ns	>0,9999	
6:WT - placebo vs. 6:WT - lithium	-1,079	-1,371 to -0,7870	Yes	****	<0,0001	
6:WT - placebo vs. 6:$2b4^{he} 2b5^{ho}$ - lithium	-0,3149	-0,6069 to -0,02297	Yes	*		0,0198
6:WT - placebo vs. 7:WT - placebo	-0,03678	-0,2568 to 0,1832	No	ns	>0,9999	
6:WT - placebo vs. 7:$2b4^{he} 2b5^{ho}$ - placebo	-0,006203	-0,2982 to 0,2858	No	ns	>0,9999	
6:WT - placebo vs. 7:WT - lithium	-1,286	-1,578 to -0,9941	Yes	****	<0,0001	
6:WT - placebo vs. 7:$2b4^{he} 2b5^{ho}$ - lithium	-0,5583	-0,8654 to -0,2511	Yes	****	<0,0001	
6:$2b4^{he} 2b5^{ho}$ - placebo vs. 6:WT - lithium	-1,069	-1,361 to -0,7771	Yes	****	<0,0001	
6:$2b4^{he} 2b5^{ho}$ - placebo vs. 6:$2b4^{he} 2b5^{ho}$ - lithium	-0,3051	-0,5970 to -0,01308	Yes	*		0,0298
6:$2b4^{he} 2b5^{ho}$ - placebo vs. 7:WT - placebo	-0,0269	-0,3189 to 0,2651	No	ns	>0,9999	
6:$2b4^{he} 2b5^{ho}$ - placebo vs. 7:$2b4^{he} 2b5^{ho}$ - placebo	0,003685	-0,2163 to 0,2237	No	ns	>0,9999	
6:$2b4^{he} 2b5^{ho}$ - placebo vs. 7:WT - lithium	-1,276	-1,568 to -0,9842	Yes	****	<0,0001	
6:$2b4^{he} 2b5^{ho}$ - placebo vs. 7:$2b4^{he} 2b5^{ho}$ - lithium	-0,5484	-0,8555 to -0,2412	Yes	****	<0,0001	
6:WT - lithium vs. 6:$2b4^{he} 2b5^{ho}$ - lithium	0,764	0,4720 to 1,056	Yes	****	<0,0001	
6:WT - lithium vs. 7:WT - placebo	1,042	0,7502 to 1,334	Yes	****	<0,0001	
6:WT - lithium vs. 7:$2b4^{he} 2b5^{ho}$ - placebo	1,073	0,7808 to 1,365	Yes	****	<0,0001	
6:WT - lithium vs. 7:WT - lithium	-0,2071	-0,4272 to 0,01291	No	ns		0,0928
6:WT - lithium vs. 7:$2b4^{he} 2b5^{ho}$ - lithium	0,5207	0,2135 to 0,8278	Yes	****	<0,0001	
6:$2b4^{he} 2b5^{ho}$ - lithium vs. 7:WT - placebo	0,2782	-0,01381 to 0,5701	No	ns		0,0834
6:$2b4^{he} 2b5^{ho}$ - lithium vs. 7:$2b4^{he} 2b5^{ho}$ - placebo	0,3087	0,01677 to 0,6007	Yes	*		0,0256
6:$2b4^{he} 2b5^{ho}$ - lithium vs. 7:WT - lithium	-0,9711	-1,263 to -0,6792	Yes	****	<0,0001	
6:$2b4^{he} 2b5^{ho}$ - lithium vs. 7:$2b4^{he} 2b5^{ho}$ - lithium	-0,2433	-0,4833 to -0,003303	Yes	*		0,0429
7:WT - placebo vs. 7:$2b4^{he} 2b5^{ho}$ - placebo	0,03058	-0,2614 to 0,3226	No	ns	>0,9999	
7:WT - placebo vs. 7:WT - lithium	-1,249	-1,541 to -0,9573	Yes	****	<0,0001	
7:WT - placebo vs. 7:$2b4^{he} 2b5^{ho}$ - lithium	-0,5215	-0,8286 to -0,2143	Yes	****	<0,0001	
7:$2b4^{he} 2b5^{ho}$ - placebo vs. 7:WT - lithium	-1,28	-1,572 to -0,9879	Yes	****	<0,0001	
7:$2b4^{he} 2b5^{ho}$ - placebo vs. 7:$2b4^{he} 2b5^{ho}$ - lithium	-0,5521	-0,8592 to -0,2449	Yes	****	<0,0001	
7:WT - lithium vs. 7:$2b4^{he} 2b5^{ho}$ - lithium	0,7278	0,4207 to 1,035	Yes	****	<0,0001	

Test details	Predicted (LS) mean 1	Predicted (LS) mean 2	Predicted (LS) mean diff,	SE of diff,	N1	N2	q	DF
1:WT - placebo vs. 1:$2b4^{he} 2b5^{ho}$ - placebo	0,1753	0,2145	-0,03916	0,07587	4	4	0,73	82
1:WT - placebo vs. 1:WT - lithium	0,1753	0,1824	-0,00706	0,07587	4	4	0,1316	82
1:WT - placebo vs. 1:$2b4^{he} 2b5^{ho}$ - lithium	0,1753	0,1436	0,0317	0,07981	4	3	0,5617	82
1:WT - placebo vs. 2:WT - placebo	0,1753	0,1706	0,004728	0,05684	4	4	0,1177	70
1:WT - placebo vs. 2:$2b4^{he} 2b5^{ho}$ - placebo	0,1753	0,1702	0,005151	0,07587	4	4	0,09601	82
1:WT - placebo vs. 2:WT - lithium	0,1753	0,2436	-0,06829	0,07587	4	4	1,273	82
1:WT - placebo vs. 2:$2b4^{he} 2b5^{ho}$ - lithium	0,1753	0,144	0,03126	0,07587	4	4	0,5827	82
1:WT - placebo vs. 3:WT - placebo	0,1753	0,1396	0,03567	0,05684	4	4	0,8875	70
1:WT - placebo vs. 3:$2b4^{he} 2b5^{ho}$ - placebo	0,1753	0,1319	0,04344	0,07587	4	4	0,8096	82
1:WT - placebo vs. 3:WT - lithium	0,1753	0,308	-0,1327	0,07587	4	4	2,474	82
1:WT - placebo vs. 3:$2b4^{he} 2b5^{ho}$ - lithium	0,1753	0,1628	0,01255	0,07587	4	4	0,234	82
1:WT - placebo vs. 4:WT - placebo	0,1753	0,1494	0,02595	0,05684	4	4	0,6456	70
1:WT - placebo vs. 4:$2b4^{he} 2b5^{ho}$ - placebo	0,1753	0,1542	0,02113	0,07587	4	4	0,3939	82
1:WT - placebo vs. 4:WT - lithium	0,1753	0,5385	-0,3632	0,07587	4	4	6,77	82
1:WT - placebo vs. 4:$2b4^{he} 2b5^{ho}$ - lithium	0,1753	0,2521	-0,07681	0,07587	4	4	1,432	82
1:WT - placebo vs. 5:WT - placebo	0,1753	0,2008	-0,02547	0,05684	4	4	0,6338	70
1:WT - placebo vs. 5:$2b4^{he} 2b5^{ho}$ - placebo	0,1753	0,1599	0,01536	0,07587	4	4	0,2863	82
1:WT - placebo vs. 5:WT - lithium	0,1753	0,929	-0,7537	0,07587	4	4	14,05	82
1:WT - placebo vs. 5:$2b4^{he} 2b5^{ho}$ - lithium	0,1753	0,3769	-0,2016	0,07587	4	4	3,758	82
1:WT - placebo vs. 6:WT - placebo	0,1753	0,1552	0,0201	0,05684	4	4	0,5002	70
1:WT - placebo vs. 6:$2b4^{he} 2b5^{ho}$ - placebo	0,1753	0,1651	0,01022	0,07587	4	4	0,1904	82
1:WT - placebo vs. 6:WT - lithium	0,1753	1,234	-1,059	0,07587	4	4	19,74	82
1:WT - placebo vs. 6:$2b4^{he} 2b5^{ho}$ - lithium	0,1753	0,4701	-0,2948	0,07587	4	4	5,496	82
1:WT - placebo vs. 7:WT - placebo	0,1753	0,192	-0,01668	0,05684	4	4	0,415	70
1:WT - placebo vs. 7:$2b4^{he} 2b5^{ho}$ - placebo	0,1753	0,1614	0,0139	0,07587	4	4	0,2591	82
1:WT - placebo vs. 7:WT - lithium	0,1753	1,441	-1,266	0,07587	4	4	23,6	82
1:WT - placebo vs. 7:$2b4^{he} 2b5^{ho}$ - lithium	0,1753	0,7135	-0,5382	0,07981	4	3	9,536	82
1:$2b4^{he} 2b5^{ho}$ - placebo vs. 1:WT - lithium	0,2145	0,1824	0,0321	0,07587	4	4	0,5984	82
1:$2b4^{he} 2b5^{ho}$ - placebo vs. 1:$2b4^{he} 2b5^{ho}$ - lithium	0,2145	0,1436	0,07086	0,07981	4	3	1,256	82
1:$2b4^{he} 2b5^{ho}$ - placebo vs. 2:WT - placebo	0,2145	0,1706	0,04389	0,07587	4	4	0,8181	82
1:$2b4^{he} 2b5^{ho}$ - placebo vs. 2:$2b4^{he} 2b5^{ho}$ - placebo	0,2145	0,1702	0,04431	0,05684	4	4	1,103	70
1:$2b4^{he} 2b5^{ho}$ - placebo vs. 2:WT - lithium	0,2145	0,2436	-0,02913	0,07587	4	4	0,543	82
1:$2b4^{he} 2b5^{ho}$ - placebo vs. 2:$2b4^{he} 2b5^{ho}$ - lithium	0,2145	0,144	0,07042	0,07587	4	4	1,313	82
1:$2b4^{he} 2b5^{ho}$ - placebo vs. 3:WT - placebo	0,2145	0,1396	0,07483	0,07587	4	4	1,395	82
1:$2b4^{he} 2b5^{ho}$ - placebo vs. 3:$2b4^{he} 2b5^{ho}$ - placebo	0,2145	0,1319	0,0826	0,05684	4	4	2,055	70
1:$2b4^{he} 2b5^{ho}$ - placebo vs. 3:WT - lithium	0,2145	0,308	-0,09358	0,07587	4	4	1,744	82
1:$2b4^{he} 2b5^{ho}$ - placebo vs. 3:$2b4^{he} 2b5^{ho}$ - lithium	0,2145	0,1628	0,05172	0,07587	4	4	0,964	82
1:$2b4^{he} 2b5^{ho}$ - placebo vs. 4:WT - placebo	0,2145	0,1494	0,06511	0,07587	4	4	1,214	82
1:$2b4^{he} 2b5^{ho}$ - placebo vs. 4:$2b4^{he} 2b5^{ho}$ - placebo	0,2145	0,1542	0,0603	0,05684	4	4	1,5	70
1:$2b4^{he} 2b5^{ho}$ - placebo vs. 4:WT - lithium	0,2145	0,5385	-0,324	0,07587	4	4	6,04	82
1:$2b4^{he} 2b5^{ho}$ - placebo vs. 4:$2b4^{he} 2b5^{ho}$ - lithium	0,2145	0,2521	-0,03764	0,07587	4	4	0,7016	82
1:$2b4^{he} 2b5^{ho}$ - placebo vs. 5:WT - placebo	0,2145	0,2008	0,01369	0,07587	4	4	0,2552	82
1:$2b4^{he} 2b5^{ho}$ - placebo vs. 5:$2b4^{he} 2b5^{ho}$ - placebo	0,2145	0,1599	0,05452	0,05684	4	4	1,357	70
1:$2b4^{he} 2b5^{ho}$ - placebo vs. 5:WT - lithium	0,2145	0,929	-0,7146	0,07587	4	4	13,32	82
1:$2b4^{he} 2b5^{ho}$ - placebo vs. 5:$2b4^{he} 2b5^{ho}$ - lithium	0,2145	0,3769	-0,1625	0,07587	4	4	3,028	82
1:$2b4^{he} 2b5^{ho}$ - placebo vs. 6:WT - placebo	0,2145	0,1552	0,05927	0,07587	4	4	1,105	82
1:$2b4^{he} 2b5^{ho}$ - placebo vs. 6:$2b4^{he} 2b5^{ho}$ - placebo	0,2145	0,1651	0,04938	0,05684	4	4	1,229	70
1:$2b4^{he} 2b5^{ho}$ - placebo vs. 6:WT - lithium	0,2145	1,234	-1,02	0,07587	4	4	19,01	82
1:$2b4^{he} 2b5^{ho}$ - placebo vs. 6:$2b4^{he} 2b5^{ho}$ - lithium	0,2145	0,4701	-0,2557	0,07587	4	4	4,766	82
1:$2b4^{he} 2b5^{ho}$ - placebo vs. 7:WT - placebo	0,2145	0,192	0,02248	0,07587	4	4	0,4191	82
1:$2b4^{he} 2b5^{ho}$ - placebo vs. 7:$2b4^{he} 2b5^{ho}$ - placebo	0,2145	0,1614	0,05306	0,05684	4	4	1,32	70
1:$2b4^{he} 2b5^{ho}$ - placebo vs. 7:WT - lithium	0,2145	1,441	-1,227	0,07587	4	4	22,87	82
1:$2b4^{he} 2b5^{ho}$ - placebo vs. 7:$2b4^{he} 2b5^{ho}$ - lithium	0,2145	0,7135	-0,499	0,07981	4	3	8,842	82
1:WT - lithium vs. 1:$2b4^{he} 2b5^{ho}$ - lithium	0,1824	0,1436	0,03876	0,07981	4	3	0,6868	82
1:WT - lithium vs. 2:WT - placebo	0,1824	0,1706	0,01179	0,07587	4	4	0,2197	82
1:WT - lithium vs. 2:$2b4^{he} 2b5^{ho}$ - placebo	0,1824	0,1702	0,01221	0,07587	4	4	0,2276	82
1:WT - lithium vs. 2:WT - lithium	0,1824	0,2436	-0,06123	0,05684	4	4	1,524	70
1:WT - lithium vs. 2:$2b4^{he} 2b5^{ho}$ - lithium	0,1824	0,144	0,03832	0,07587	4	4	0,7143	82
1:WT - lithium vs. 3:WT - placebo	0,1824	0,1396	0,04273	0,07587	4	4	0,7964	82
1:WT - lithium vs. 3:$2b4^{he} 2b5^{ho}$ - placebo	0,1824	0,1319	0,05049	0,07587	4	4	0,9412	82
1:WT - lithium vs. 3:WT - lithium	0,1824	0,308	-0,1257	0,05684	4	4	3,127	70
1:WT - lithium vs. 3:$2b4^{he} 2b5^{ho}$ - lithium	0,1824	0,1628	0,01961	0,07587	4	4	0,3656	82
1:WT - lithium vs. 4:WT - placebo	0,1824	0,1494	0,03301	0,07587	4	4	0,6152	82
1:WT - lithium vs. 4:$2b4^{he} 2b5^{ho}$ - placebo	0,1824	0,1542	0,02819	0,07587	4	4	0,5255	82
1:WT - lithium vs. 4:WT - lithium	0,1824	0,5385	-0,3561	0,05684	4	4	8,861	70
1:WT - lithium vs. 4:$2b4^{he} 2b5^{ho}$ - lithium	0,1824	0,2521	-0,06975	0,07587	4	4	1,3	82
1:WT - lithium vs. 5:WT - placebo	0,1824	0,2008	-0,01841	0,07587	4	4	0,3432	82
1:WT - lithium vs. 5:$2b4^{he} 2b5^{ho}$ - placebo	0,1824	0,1599	0,02242	0,07587	4	4	0,4179	82
1:WT - lithium vs. 5:WT - lithium	0,1824	0,929	-0,7467	0,05684	4	4	18,58	70
1:WT - lithium vs. 5:$2b4^{he} 2b5^{ho}$ - lithium	0,1824	0,3769	-0,1946	0,07587	4	4	3,627	82
1:WT - lithium vs. 6:WT - placebo	0,1824	0,1552	0,02716	0,07587	4	4	0,5063	82
1:WT - lithium vs. 6:$2b4^{he} 2b5^{ho}$ - placebo	0,1824	0,1651	0,01728	0,07587	4	4	0,322	82
1:WT - lithium vs. 6:WT - lithium	0,1824	1,234	-1,052	0,05684	4	4	26,17	70
1:WT - lithium vs. 6:$2b4^{he} 2b5^{ho}$ - lithium	0,1824	0,4701	-0,2878	0,07587	4	4	5,364	82
1:WT - lithium vs. 7:WT - placebo	0,1824	0,192	-0,00962	0,07587	4	4	0,1793	82
1:WT - lithium vs. 7:$2b4^{he} 2b5^{ho}$ - placebo	0,1824	0,1614	0,02096	0,07587	4	4	0,3907	82
1:WT - lithium vs. 7:WT - lithium	0,1824	1,441	-1,259	0,05684	4	4	31,32	70
1:WT - lithium vs. 7:$2b4^{he} 2b5^{ho}$ - lithium	0,1824	0,7135	-0,5311	0,07981	4	3	9,411	82
1:$2b4^{he} 2b5^{ho}$ - lithium vs. 2:WT - placebo	0,1436	0,1706	-0,02697	0,07981	3	4	0,4779	82
1:$2b4^{he} 2b5^{ho}$ - lithium vs. 2:$2b4^{he} 2b5^{ho}$ - placebo	0,1436	0,1702	-0,02655	0,07981	3	4	0,4704	82
1:$2b4^{he} 2b5^{ho}$ - lithium vs. 2:WT - lithium	0,1436	0,2436	-0,09999	0,07981	3	4	1,772	82
1:$2b4^{he} 2b5^{ho}$ - lithium vs. 2:$2b4^{he} 2b5^{ho}$ - lithium	0,1436	0,144	-0,0004402	0,062	3	4	0,01004	70
1:$2b4^{he} 2b5^{ho}$ - lithium vs. 3:WT - placebo	0,1436	0,1396	0,003967	0,07981	3	4	0,07029	82
1:$2b4^{he} 2b5^{ho}$ - lithium vs. 3:$2b4^{he} 2b5^{ho}$ - placebo	0,1436	0,1319	0,01173	0,07981	3	4	0,2079	82
1:$2b4^{he} 2b5^{ho}$ - lithium vs. 3:WT - lithium	0,1436	0,308	-0,1644	0,07981	3	4	2,914	82
1:$2b4^{he} 2b5^{ho}$ - lithium vs. 3:$2b4^{he} 2b5^{ho}$ - lithium	0,1436	0,1628	-0,01915	0,062	3	4	0,4367	70
1:$2b4^{he} 2b5^{ho}$ - lithium vs. 4:WT - placebo	0,1436	0,1494	-0,005755	0,07981	3	4	0,102	82
1:$2b4^{he} 2b5^{ho}$ - lithium vs. 4:$2b4^{he} 2b5^{ho}$ - placebo	0,1436	0,1542	-0,01057	0,07981	3	4	0,1872	82
1:$2b4^{he} 2b5^{ho}$ - lithium vs. 4:WT - lithium	0,1436	0,5385	-0,3949	0,07981	3	4	6,997	82
1:$2b4^{he} 2b5^{ho}$ - lithium vs. 4:$2b4^{he} 2b5^{ho}$ - lithium	0,1436	0,2521	-0,1085	0,062	3	4	2,475	70
1:$2b4^{he} 2b5^{ho}$ - lithium vs. 5:WT - placebo	0,1436	0,2008	-0,05717	0,07981	3	4	1,013	82
1:$2b4^{he} 2b5^{ho}$ - lithium vs. 5:$2b4^{he} 2b5^{ho}$ - placebo	0,1436	0,1599	-0,01634	0,07981	3	4	0,2896	82
1:$2b4^{he} 2b5^{ho}$ - lithium vs. 5:WT - lithium	0,1436	0,929	-0,7854	0,07981	3	4	13,92	82
1:$2b4^{he} 2b5^{ho}$ - lithium vs. 5:$2b4^{he} 2b5^{ho}$ - lithium	0,1436	0,3769	-0,2333	0,062	3	4	5,322	70
1:$2b4^{he} 2b5^{ho}$ - lithium vs. 6:WT - placebo	0,1436	0,1552	-0,0116	0,07981	3	4	0,2055	82
1:$2b4^{he} 2b5^{ho}$ - lithium vs. 6:$2b4^{he} 2b5^{ho}$ - placebo	0,1436	0,1651	-0,02148	0,07981	3	4	0,3807	82
1:$2b4^{he} 2b5^{ho}$ - lithium vs. 6:WT - lithium	0,1436	1,234	-1,091	0,07981	3	4	19,32	82
1:$2b4^{he} 2b5^{ho}$ - lithium vs. 6:$2b4^{he} 2b5^{ho}$ - lithium	0,1436	0,4701	-0,3265	0,062	3	4	7,448	70
1:$2b4^{he} 2b5^{ho}$ - lithium vs. 7:WT - placebo	0,1436	0,192	-0,04838	0,07981	3	4	0,8573	82
1:$2b4^{he} 2b5^{ho}$ - lithium vs. 7:$2b4^{he} 2b5^{ho}$ - placebo	0,1436	0,1614	-0,0178	0,07981	3	4	0,3154	82
1:$2b4^{he} 2b5^{ho}$ - lithium vs. 7:WT - lithium	0,1436	1,441	-1,298	0,07981	3	4	22,99	82
1:$2b4^{he} 2b5^{ho}$ - lithium vs. 7:$2b4^{he} 2b5^{ho}$ - lithium	0,1436	0,7135	-0,5699	0,06719	3	3	12	70
2:WT - placebo vs. 2:$2b4^{he} 2b5^{ho}$ - placebo	0,1706	0,1702	0,0004225	0,07587	4	4	0,007875	82
2:WT - placebo vs. 2:WT - lithium	0,1706	0,2436	-0,07302	0,07587	4	4	1,361	82
2:WT - placebo vs. 2:$2b4^{he} 2b5^{ho}$ - lithium	0,1706	0,144	0,02653	0,07587	4	4	0,4946	82
2:WT - placebo vs. 3:WT - placebo	0,1706	0,1396	0,03094	0,05684	4	4	0,7698	70
2:WT - placebo vs. 3:$2b4^{he} 2b5^{ho}$ - placebo	0,1706	0,1319	0,03871	0,07587	4	4	0,7215	82
2:WT - placebo vs. 3:WT - lithium	0,1706	0,308	-0,1375	0,07587	4	4	2,562	82
2:WT - placebo vs. 3:$2b4^{he} 2b5^{ho}$ - lithium	0,1706	0,1628	0,007826	0,07587	4	4	0,1459	82
2:WT - placebo vs. 4:WT - placebo	0,1706	0,1494	0,02122	0,05684	4	4	0,5279	70
2:WT - placebo vs. 4:$2b4^{he} 2b5^{ho}$ - placebo	0,1706	0,1542	0,01641	0,07587	4	4	0,3058	82
2:WT - placebo vs. 4:WT - lithium	0,1706	0,5385	-0,3679	0,07587	4	4	6,858	82
2:WT - placebo vs. 4:$2b4^{he} 2b5^{ho}$ - lithium	0,1706	0,2521	-0,08153	0,07587	4	4	1,52	82
2:WT - placebo vs. 5:WT - placebo	0,1706	0,2008	-0,0302	0,05684	4	4	0,7515	70
2:WT - placebo vs. 5:$2b4^{he} 2b5^{ho}$ - placebo	0,1706	0,1599	0,01063	0,07587	4	4	0,1981	82
2:WT - placebo vs. 5:WT - lithium	0,1706	0,929	-0,7585	0,07587	4	4	14,14	82
2:WT - placebo vs. 5:$2b4^{he} 2b5^{ho}$ - lithium	0,1706	0,3769	-0,2064	0,07587	4	4	3,846	82
2:WT - placebo vs. 6:WT - placebo	0,1706	0,1552	0,01538	0,05684	4	4	0,3826	70
2:WT - placebo vs. 6:$2b4^{he} 2b5^{ho}$ - placebo	0,1706	0,1651	0,005488	0,07587	4	4	0,1023	82
2:WT - placebo vs. 6:WT - lithium	0,1706	1,234	-1,064	0,07587	4	4	19,82	82
2:WT - placebo vs. 6:$2b4^{he} 2b5^{ho}$ - lithium	0,1706	0,4701	-0,2996	0,07587	4	4	5,584	82
2:WT - placebo vs. 7:WT - placebo	0,1706	0,192	-0,02141	0,05684	4	4	0,5327	70
2:WT - placebo vs. 7:$2b4^{he} 2b5^{ho}$ - placebo	0,1706	0,1614	0,009173	0,07587	4	4	0,171	82
2:WT - placebo vs. 7:WT - lithium	0,1706	1,441	-1,271	0,07587	4	4	23,69	82
2:WT - placebo vs. 7:$2b4^{he} 2b5^{ho}$ - lithium	0,1706	0,7135	-0,5429	0,07981	4	3	9,62	82
2:$2b4^{he} 2b5^{ho}$ - placebo vs. 2:WT - lithium	0,1702	0,2436	-0,07344	0,07587	4	4	1,369	82
2:$2b4^{he} 2b5^{ho}$ - placebo vs. 2:$2b4^{he} 2b5^{ho}$ - lithium	0,1702	0,144	0,02611	0,07587	4	4	0,4867	82
2:$2b4^{he} 2b5^{ho}$ - placebo vs. 3:WT - placebo	0,1702	0,1396	0,03052	0,07587	4	4	0,5688	82
2:$2b4^{he} 2b5^{ho}$ - placebo vs. 3:$2b4^{he} 2b5^{ho}$ - placebo	0,1702	0,1319	0,03828	0,05684	4	4	0,9526	70
2:$2b4^{he} 2b5^{ho}$ - placebo vs. 3:WT - lithium	0,1702	0,308	-0,1379	0,07587	4	4	2,57	82
2:$2b4^{he} 2b5^{ho}$ - placebo vs. 3:$2b4^{he} 2b5^{ho}$ - lithium	0,1702	0,1628	0,007403	0,07587	4	4	0,138	82
2:$2b4^{he} 2b5^{ho}$ - placebo vs. 4:WT - placebo	0,1702	0,1494	0,0208	0,07587	4	4	0,3876	82
2:$2b4^{he} 2b5^{ho}$ - placebo vs. 4:$2b4^{he} 2b5^{ho}$ - placebo	0,1702	0,1542	0,01598	0,05684	4	4	0,3977	70
2:$2b4^{he} 2b5^{ho}$ - placebo vs. 4:WT - lithium	0,1702	0,5385	-0,3683	0,07587	4	4	6,866	82
2:$2b4^{he} 2b5^{ho}$ - placebo vs. 4:$2b4^{he} 2b5^{ho}$ - lithium	0,1702	0,2521	-0,08196	0,07587	4	4	1,528	82
2:$2b4^{he} 2b5^{ho}$ - placebo vs. 5:WT - placebo	0,1702	0,2008	-0,03062	0,07587	4	4	0,5708	82
2:$2b4^{he} 2b5^{ho}$ - placebo vs. 5:$2b4^{he} 2b5^{ho}$ - placebo	0,1702	0,1599	0,01021	0,05684	4	4	0,254	70
2:$2b4^{he} 2b5^{ho}$ - placebo vs. 5:WT - lithium	0,1702	0,929	-0,7589	0,07587	4	4	14,15	82
2:$2b4^{he} 2b5^{ho}$ - placebo vs. 5:$2b4^{he} 2b5^{ho}$ - lithium	0,1702	0,3769	-0,2068	0,07587	4	4	3,854	82
2:$2b4^{he} 2b5^{ho}$ - placebo vs. 6:WT - placebo	0,1702	0,1552	0,01495	0,07587	4	4	0,2787	82
2:$2b4^{he} 2b5^{ho}$ - placebo vs. 6:$2b4^{he} 2b5^{ho}$ - placebo	0,1702	0,1651	0,005065	0,05684	4	4	0,126	70
2:$2b4^{he} 2b5^{ho}$ - placebo vs. 6:WT - lithium	0,1702	1,234	-1,064	0,07587	4	4	19,83	82
2:$2b4^{he} 2b5^{ho}$ - placebo vs. 6:$2b4^{he} 2b5^{ho}$ - lithium	0,1702	0,4701	-0,3	0,07587	4	4	5,592	82
2:$2b4^{he} 2b5^{ho}$ - placebo vs. 7:WT - placebo	0,1702	0,192	-0,02183	0,07587	4	4	0,4069	82
2:$2b4^{he} 2b5^{ho}$ - placebo vs. 7:$2b4^{he} 2b5^{ho}$ - placebo	0,1702	0,1614	0,00875	0,05684	4	4	0,2177	70
2:$2b4^{he} 2b5^{ho}$ - placebo vs. 7:WT - lithium	0,1702	1,441	-1,271	0,07587	4	4	23,69	82
2:$2b4^{he} 2b5^{ho}$ - placebo vs. 7:$2b4^{he} 2b5^{ho}$ - lithium	0,1702	0,7135	-0,5433	0,07981	4	3	9,627	82
2:WT - lithium vs. 2:$2b4^{he} 2b5^{ho}$ - lithium	0,2436	0,144	0,09955	0,07587	4	4	1,856	82
2:WT - lithium vs. 3:WT - placebo	0,2436	0,1396	0,104	0,07587	4	4	1,938	82
2:WT - lithium vs. 3:$2b4^{he} 2b5^{ho}$ - placebo	0,2436	0,1319	0,1117	0,07587	4	4	2,083	82
2:WT - lithium vs. 3:WT - lithium	0,2436	0,308	-0,06445	0,05684	4	4	1,604	70
2:WT - lithium vs. 3:$2b4^{he} 2b5^{ho}$ - lithium	0,2436	0,1628	0,08085	0,07587	4	4	1,507	82
2:WT - lithium vs. 4:WT - placebo	0,2436	0,1494	0,09424	0,07587	4	4	1,757	82
2:WT - lithium vs. 4:$2b4^{he} 2b5^{ho}$ - placebo	0,2436	0,1542	0,08943	0,07587	4	4	1,667	82
2:WT - lithium vs. 4:WT - lithium	0,2436	0,5385	-0,2949	0,05684	4	4	7,337	70
2:WT - lithium vs. 4:$2b4^{he} 2b5^{ho}$ - lithium	0,2436	0,2521	-0,008511	0,07587	4	4	0,1587	82
2:WT - lithium vs. 5:WT - placebo	0,2436	0,2008	0,04282	0,07587	4	4	0,7982	82
2:WT - lithium vs. 5:$2b4^{he} 2b5^{ho}$ - placebo	0,2436	0,1599	0,08365	0,07587	4	4	1,559	82
2:WT - lithium vs. 5:WT - lithium	0,2436	0,929	-0,6854	0,05684	4	4	17,06	70
2:WT - lithium vs. 5:$2b4^{he} 2b5^{ho}$ - lithium	0,2436	0,3769	-0,1333	0,07587	4	4	2,485	82
2:WT - lithium vs. 6:WT - placebo	0,2436	0,1552	0,0884	0,07587	4	4	1,648	82
2:WT - lithium vs. 6:$2b4^{he} 2b5^{ho}$ - placebo	0,2436	0,1651	0,07851	0,07587	4	4	1,463	82
2:WT - lithium vs. 6:WT - lithium	0,2436	1,234	-0,9906	0,05684	4	4	24,65	70
2:WT - lithium vs. 6:$2b4^{he} 2b5^{ho}$ - lithium	0,2436	0,4701	-0,2265	0,07587	4	4	4,223	82
2:WT - lithium vs. 7:WT - placebo	0,2436	0,192	0,05161	0,07587	4	4	0,9621	82
2:WT - lithium vs. 7:$2b4^{he} 2b5^{ho}$ - placebo	0,2436	0,1614	0,08219	0,07587	4	4	1,532	82
2:WT - lithium vs. 7:WT - lithium	0,2436	1,441	-1,198	0,05684	4	4	29,8	70
2:WT - lithium vs. 7:$2b4^{he} 2b5^{ho}$ - lithium	0,2436	0,7135	-0,4699	0,07981	4	3	8,326	82
2:$2b4^{he} 2b5^{ho}$ - lithium vs. 3:WT - placebo	0,144	0,1396	0,004407	0,07587	4	4	0,08215	82
2:$2b4^{he} 2b5^{ho}$ - lithium vs. 3:$2b4^{he} 2b5^{ho}$ - placebo	0,144	0,1319	0,01217	0,07587	4	4	0,2269	82
2:$2b4^{he} 2b5^{ho}$ - lithium vs. 3:WT - lithium	0,144	0,308	-0,164	0,07587	4	4	3,057	82
2:$2b4^{he} 2b5^{ho}$ - lithium vs. 3:$2b4^{he} 2b5^{ho}$ - lithium	0,144	0,1628	-0,01871	0,05684	4	4	0,4655	70
2:$2b4^{he} 2b5^{ho}$ - lithium vs. 4:WT - placebo	0,144	0,1494	-0,005315	0,07587	4	4	0,09906	82
2:$2b4^{he} 2b5^{ho}$ - lithium vs. 4:$2b4^{he} 2b5^{ho}$ - placebo	0,144	0,1542	-0,01013	0,07587	4	4	0,1887	82
2:$2b4^{he} 2b5^{ho}$ - lithium vs. 4:WT - lithium	0,144	0,5385	-0,3944	0,07587	4	4	7,352	82
2:$2b4^{he} 2b5^{ho}$ - lithium vs. 4:$2b4^{he} 2b5^{ho}$ - lithium	0,144	0,2521	-0,1081	0,05684	4	4	2,689	70
2:$2b4^{he} 2b5^{ho}$ - lithium vs. 5:WT - placebo	0,144	0,2008	-0,05673	0,07587	4	4	1,057	82
2:$2b4^{he} 2b5^{ho}$ - lithium vs. 5:$2b4^{he} 2b5^{ho}$ - placebo	0,144	0,1599	-0,0159	0,07587	4	4	0,2964	82
2:$2b4^{he} 2b5^{ho}$ - lithium vs. 5:WT - lithium	0,144	0,929	-0,785	0,07587	4	4	14,63	82
2:$2b4^{he} 2b5^{ho}$ - lithium vs. 5:$2b4^{he} 2b5^{ho}$ - lithium	0,144	0,3769	-0,2329	0,05684	4	4	5,795	70
2:$2b4^{he} 2b5^{ho}$ - lithium vs. 6:WT - placebo	0,144	0,1552	-0,01116	0,07587	4	4	0,208	82
2:$2b4^{he} 2b5^{ho}$ - lithium vs. 6:$2b4^{he} 2b5^{ho}$ - placebo	0,144	0,1651	-0,02104	0,07587	4	4	0,3923	82
2:$2b4^{he} 2b5^{ho}$ - lithium vs. 6:WT - lithium	0,144	1,234	-1,09	0,07587	4	4	20,32	82
2:$2b4^{he} 2b5^{ho}$ - lithium vs. 6:$2b4^{he} 2b5^{ho}$ - lithium	0,144	0,4701	-0,3261	0,05684	4	4	8,114	70
2:$2b4^{he} 2b5^{ho}$ - lithium vs. 7:WT - placebo	0,144	0,192	-0,04794	0,07587	4	4	0,8936	82
2:$2b4^{he} 2b5^{ho}$ - lithium vs. 7:$2b4^{he} 2b5^{ho}$ - placebo	0,144	0,1614	-0,01736	0,07587	4	4	0,3236	82
2:$2b4^{he} 2b5^{ho}$ - lithium vs. 7:WT - lithium	0,144	1,441	-1,297	0,07587	4	4	24,18	82
2:$2b4^{he} 2b5^{ho}$ - lithium vs. 7:$2b4^{he} 2b5^{ho}$ - lithium	0,144	0,7135	-0,5694	0,062	4	3	12,99	70

3:WT - placebo vs. 3:$2b4^{he} 2b5^{ho}$ - placebo	0,1396	0,1319	0,007767	0,07587	4	4	0,1448	82
3:WT - placebo vs. 3:WT - lithium	0,1396	0,308	-0,1684	0,07587	4	4	3,139	82
3:WT - placebo vs. 3:$2b4^{he} 2b5^{ho}$ - lithium	0,1396	0,1628	-0,02311	0,07587	4	4	0,4308	82
3:WT - placebo vs. 4:WT - placebo	0,1396	0,1494	-0,009722	0,05684	4	4	0,2419	70
3:WT - placebo vs. 4:$2b4^{he} 2b5^{ho}$ - placebo	0,1396	0,1542	-0,01453	0,07587	4	4	0,2709	82
3:WT - placebo vs. 4:WT - lithium	0,1396	0,5385	-0,3988	0,07587	4	4	7,434	82
3:WT - placebo vs. 4:$2b4^{he} 2b5^{ho}$ - lithium	0,1396	0,2521	-0,1125	0,07587	4	4	2,096	82
3:WT - placebo vs. 5:WT - placebo	0,1396	0,2008	-0,06114	0,05684	4	4	1,521	70
3:WT - placebo vs. 5:$2b4^{he} 2b5^{ho}$ - placebo	0,1396	0,1599	-0,02031	0,07587	4	4	0,3786	82
3:WT - placebo vs. 5:WT - lithium	0,1396	0,929	-0,7894	0,07587	4	4	14,71	82
3:WT - placebo vs. 5:$2b4^{he} 2b5^{ho}$ - lithium	0,1396	0,3769	-0,2373	0,07587	4	4	4,423	82
3:WT - placebo vs. 6:WT - placebo	0,1396	0,1552	-0,01556	0,05684	4	4	0,3873	70
3:WT - placebo vs. 6:$2b4^{he} 2b5^{ho}$ - placebo	0,1396	0,1651	-0,02545	0,07587	4	4	0,4744	82
3:WT - placebo vs. 6:WT - lithium	0,1396	1,234	-1,095	0,07587	4	4	20,4	82
3:WT - placebo vs. 6:$2b4^{he} 2b5^{ho}$ - lithium	0,1396	0,4701	-0,3305	0,07587	4	4	6,161	82
3:WT - placebo vs. 7:WT - placebo	0,1396	0,192	-0,05235	0,05684	4	4	1,303	70
3:WT - placebo vs. 7:$2b4^{he} 2b5^{ho}$ - placebo	0,1396	0,1614	-0,02177	0,07587	4	4	0,4057	82
3:WT - placebo vs. 7:WT - lithium	0,1396	1,441	-1,302	0,07587	4	4	24,26	82
3:WT - placebo vs. 7:$2b4^{he} 2b5^{ho}$ - lithium	0,1396	0,7135	-0,5738	0,07981	4	3	10,17	82
3:$2b4^{he} 2b5^{ho}$ - placebo vs. 3:WT - lithium	0,1319	0,308	-0,1762	0,07587	4	4	3,284	82
3:$2b4^{he} 2b5^{ho}$ - placebo vs. 3:$2b4^{he} 2b5^{ho}$ - lithium	0,1319	0,1628	-0,03088	0,07587	4	4	0,5756	82
3:$2b4^{he} 2b5^{ho}$ - placebo vs. 4:WT - placebo	0,1319	0,1494	-0,01749	0,07587	4	4	0,326	82
3:$2b4^{he} 2b5^{ho}$ - placebo vs. 4:$2b4^{he} 2b5^{ho}$ - placebo	0,1319	0,1542	-0,0223	0,05684	4	4	0,5549	70
3:$2b4^{he} 2b5^{ho}$ - placebo vs. 4:WT - lithium	0,1319	0,5385	-0,4066	0,07587	4	4	7,579	82
3:$2b4^{he} 2b5^{ho}$ - placebo vs. 4:$2b4^{he} 2b5^{ho}$ - lithium	0,1319	0,2521	-0,1202	0,07587	4	4	2,241	82
3:$2b4^{he} 2b5^{ho}$ - placebo vs. 5:WT - placebo	0,1319	0,2008	-0,06891	0,07587	4	4	1,284	82
3:$2b4^{he} 2b5^{ho}$ - placebo vs. 5:$2b4^{he} 2b5^{ho}$ - placebo	0,1319	0,1599	-0,02808	0,05684	4	4	0,6986	70
3:$2b4^{he} 2b5^{ho}$ - placebo vs. 5:WT - lithium	0,1319	0,929	-0,7972	0,07587	4	4	14,86	82
3:$2b4^{he} 2b5^{ho}$ - placebo vs. 5:$2b4^{he} 2b5^{ho}$ - lithium	0,1319	0,3769	-0,2451	0,07587	4	4	4,568	82
3:$2b4^{he} 2b5^{ho}$ - placebo vs. 6:WT - placebo	0,1319	0,1552	-0,02333	0,07587	4	4	0,4349	82
3:$2b4^{he} 2b5^{ho}$ - placebo vs. 6:$2b4^{he} 2b5^{ho}$ - placebo	0,1319	0,1651	-0,03322	0,05684	4	4	0,8266	70
3:$2b4^{he} 2b5^{ho}$ - placebo vs. 6:WT - lithium	0,1319	1,234	-1,102	0,07587	4	4	20,55	82
3:$2b4^{he} 2b5^{ho}$ - placebo vs. 6:$2b4^{he} 2b5^{ho}$ - lithium	0,1319	0,4701	-0,3383	0,07587	4	4	6,305	82
3:$2b4^{he} 2b5^{ho}$ - placebo vs. 7:WT - placebo	0,1319	0,192	-0,06011	0,07587	4	4	1,121	82
3:$2b4^{he} 2b5^{ho}$ - placebo vs. 7:$2b4^{he} 2b5^{ho}$ - placebo	0,1319	0,1614	-0,02953	0,05684	4	4	0,7349	70
3:$2b4^{he} 2b5^{ho}$ - placebo vs. 7:WT - lithium	0,1319	1,441	-1,309	0,07587	4	4	24,41	82
3:$2b4^{he} 2b5^{ho}$ - placebo vs. 7:$2b4^{he} 2b5^{ho}$ - lithium	0,1319	0,7135	-0,5816	0,07981	4	3	10,31	82
3:WT - lithium vs. 3:$2b4^{he} 2b5^{ho}$ - lithium	0,308	0,1628	0,1453	0,07587	4	4	2,708	82
3:WT - lithium vs. 4:WT - placebo	0,308	0,1494	0,1587	0,07587	4	4	2,958	82
3:WT - lithium vs. 4:$2b4^{he} 2b5^{ho}$ - placebo	0,308	0,1542	0,1539	0,07587	4	4	2,868	82
3:WT - lithium vs. 4:WT - lithium	0,308	0,5385	-0,2304	0,05684	4	4	5,734	70
3:WT - lithium vs. 4:$2b4^{he} 2b5^{ho}$ - lithium	0,308	0,2521	0,05594	0,07587	4	4	1,043	82
3:WT - lithium vs. 5:WT - placebo	0,308	0,2008	0,1073	0,07587	4	4	1,999	82
3:WT - lithium vs. 5:$2b4^{he} 2b5^{ho}$ - placebo	0,308	0,1599	0,1481	0,07587	4	4	2,761	82
3:WT - lithium vs. 5:WT - lithium	0,308	0,929	-0,621	0,05684	4	4	15,45	70
3:WT - lithium vs. 5:$2b4^{he} 2b5^{ho}$ - lithium	0,308	0,3769	-0,06888	0,07587	4	4	1,284	82
3:WT - lithium vs. 6:WT - placebo	0,308	0,1552	0,1528	0,07587	4	4	2,849	82
3:WT - lithium vs. 6:$2b4^{he} 2b5^{ho}$ - placebo	0,308	0,1651	0,143	0,07587	4	4	2,665	82
3:WT - lithium vs. 6:WT - lithium	0,308	1,234	-0,9261	0,05684	4	4	23,04	70
3:WT - lithium vs. 6:$2b4^{he} 2b5^{ho}$ - lithium	0,308	0,4701	-0,1621	0,07587	4	4	3,021	82
3:WT - lithium vs. 7:WT - placebo	0,308	0,192	0,1161	0,07587	4	4	2,163	82
3:WT - lithium vs. 7:$2b4^{he} 2b5^{ho}$ - placebo	0,308	0,1614	0,1466	0,07587	4	4	2,733	82
3:WT - lithium vs. 7:WT - lithium	0,308	1,441	-1,133	0,05684	4	4	28,2	70
3:WT - lithium vs. 7:$2b4^{he} 2b5^{ho}$ - lithium	0,308	0,7135	-0,4054	0,07981	4	3	7,184	82
3:$2b4^{he} 2b5^{ho}$ - lithium vs. 4:WT - placebo	0,1628	0,1494	0,01339	0,07587	4	4	0,2496	82
3:$2b4^{he} 2b5^{ho}$ - lithium vs. 4:$2b4^{he} 2b5^{ho}$ - placebo	0,1628	0,1542	0,008581	0,07587	4	4	0,1599	82
3:$2b4^{he} 2b5^{ho}$ - lithium vs. 4:WT - lithium	0,1628	0,5385	-0,3757	0,07587	4	4	7,004	82
3:$2b4^{he} 2b5^{ho}$ - lithium vs. 4:$2b4^{he} 2b5^{ho}$ - lithium	0,1628	0,2521	-0,08936	0,05684	4	4	2,223	70
3:$2b4^{he} 2b5^{ho}$ - lithium vs. 5:WT - placebo	0,1628	0,2008	-0,03803	0,07587	4	4	0,7088	82
3:$2b4^{he} 2b5^{ho}$ - lithium vs. 5:$2b4^{he} 2b5^{ho}$ - placebo	0,1628	0,1599	0,002803	0,07587	4	4	0,05226	82
3:$2b4^{he} 2b5^{ho}$ - lithium vs. 5:WT - lithium	0,1628	0,929	-0,7663	0,07587	4	4	14,28	82
3:$2b4^{he} 2b5^{ho}$ - lithium vs. 5:$2b4^{he} 2b5^{ho}$ - lithium	0,1628	0,3769	-0,2142	0,05684	4	4	5,329	70
3:$2b4^{he} 2b5^{ho}$ - lithium vs. 6:WT - placebo	0,1628	0,1552	0,00755	0,07587	4	4	0,1407	82
3:$2b4^{he} 2b5^{ho}$ - lithium vs. 6:$2b4^{he} 2b5^{ho}$ - placebo	0,1628	0,1651	-0,002338	0,07587	4	4	0,04358	82
3:$2b4^{he} 2b5^{ho}$ - lithium vs. 6:WT - lithium	0,1628	1,234	-1,071	0,07587	4	4	19,97	82
3:$2b4^{he} 2b5^{ho}$ - lithium vs. 6:$2b4^{he} 2b5^{ho}$ - lithium	0,1628	0,4701	-0,3074	0,05684	4	4	7,649	70
3:$2b4^{he} 2b5^{ho}$ - lithium vs. 7:WT - placebo	0,1628	0,192	-0,02923	0,07587	4	4	0,5449	82
3:$2b4^{he} 2b5^{ho}$ - lithium vs. 7:$2b4^{he} 2b5^{ho}$ - placebo	0,1628	0,1614	0,001347	0,07587	4	4	0,02511	82
3:$2b4^{he} 2b5^{ho}$ - lithium vs. 7:WT - lithium	0,1628	1,441	-1,279	0,07587	4	4	23,83	82
3:$2b4^{he} 2b5^{ho}$ - lithium vs. 7:$2b4^{he} 2b5^{ho}$ - lithium	0,1628	0,7135	-0,5507	0,062	4	3	12,56	70
4:WT - placebo vs. 4:$2b4^{he} 2b5^{ho}$ - placebo	0,1494	0,1542	-0,004811	0,07587	4	4	0,08969	82
4:WT - placebo vs. 4:WT - lithium	0,1494	0,5385	-0,3891	0,07587	4	4	7,253	82
4:WT - placebo vs. 4:$2b4^{he} 2b5^{ho}$ - lithium	0,1494	0,2521	-0,1028	0,07587	4	4	1,915	82
4:WT - placebo vs. 5:WT - placebo	0,1494	0,2008	-0,05142	0,05684	4	4	1,279	70
4:WT - placebo vs. 5:$2b4^{he} 2b5^{ho}$ - placebo	0,1494	0,1599	-0,01059	0,07587	4	4	0,1974	82
4:WT - placebo vs. 5:WT - lithium	0,1494	0,929	-0,7797	0,07587	4	4	14,53	82
4:WT - placebo vs. 5:$2b4^{he} 2b5^{ho}$ - lithium	0,1494	0,3769	-0,2276	0,07587	4	4	4,242	82
4:WT - placebo vs. 6:WT - placebo	0,1494	0,1552	-0,005842	0,05684	4	4	0,1454	70
4:WT - placebo vs. 6:$2b4^{he} 2b5^{ho}$ - placebo	0,1494	0,1651	-0,01573	0,07587	4	4	0,2932	82
4:WT - placebo vs. 6:WT - lithium	0,1494	1,234	-1,085	0,07587	4	4	20,22	82
4:WT - placebo vs. 6:$2b4^{he} 2b5^{ho}$ - lithium	0,1494	0,4701	-0,3208	0,07587	4	4	5,979	82
4:WT - placebo vs. 7:WT - placebo	0,1494	0,192	-0,04263	0,05684	4	4	1,061	70
4:WT - placebo vs. 7:$2b4^{he} 2b5^{ho}$ - placebo	0,1494	0,1614	-0,01205	0,07587	4	4	0,2245	82
4:WT - placebo vs. 7:WT - lithium	0,1494	1,441	-1,292	0,07587	4	4	24,08	82
4:WT - placebo vs. 7:$2b4^{he} 2b5^{ho}$ - lithium	0,1494	0,7135	-0,5641	0,07981	4	3	9,996	82
4:$2b4^{he} 2b5^{ho}$ - placebo vs. 4:WT - lithium	0,1542	0,5385	-0,3843	0,07587	4	4	7,163	82
4:$2b4^{he} 2b5^{ho}$ - placebo vs. 4:$2b4^{he} 2b5^{ho}$ - lithium	0,1542	0,2521	-0,09794	0,07587	4	4	1,826	82
4:$2b4^{he} 2b5^{ho}$ - placebo vs. 5:WT - placebo	0,1542	0,2008	-0,04661	0,07587	4	4	0,8688	82
4:$2b4^{he} 2b5^{ho}$ - placebo vs. 5:$2b4^{he} 2b5^{ho}$ - placebo	0,1542	0,1599	-0,005777	0,05684	4	4	0,1438	70
4:$2b4^{he} 2b5^{ho}$ - placebo vs. 5:WT - lithium	0,1542	0,929	-0,7749	0,07587	4	4	14,44	82
4:$2b4^{he} 2b5^{ho}$ - placebo vs. 5:$2b4^{he} 2b5^{ho}$ - lithium	0,1542	0,3769	-0,2228	0,07587	4	4	4,152	82
4:$2b4^{he} 2b5^{ho}$ - placebo vs. 6:WT - placebo	0,1542	0,1552	-0,001031	0,07587	4	4	0,01922	82
4:$2b4^{he} 2b5^{ho}$ - placebo vs. 6:$2b4^{he} 2b5^{ho}$ - placebo	0,1542	0,1651	-0,01092	0,05684	4	4	0,2717	70
4:$2b4^{he} 2b5^{ho}$ - placebo vs. 6:WT - lithium	0,1542	1,234	-1,08	0,07587	4	4	20,13	82
4:$2b4^{he} 2b5^{ho}$ - placebo vs. 6:$2b4^{he} 2b5^{ho}$ - lithium	0,1542	0,4701	-0,316	0,07587	4	4	5,89	82
4:$2b4^{he} 2b5^{ho}$ - placebo vs. 7:WT - placebo	0,1542	0,192	-0,03781	0,07587	4	4	0,7048	82
4:$2b4^{he} 2b5^{ho}$ - placebo vs. 7:$2b4^{he} 2b5^{ho}$ - placebo	0,1542	0,1614	-0,007234	0,05684	4	4	0,18	70
4:$2b4^{he} 2b5^{ho}$ - placebo vs. 7:WT - lithium	0,1542	1,441	-1,287	0,07587	4	4	23,99	82
4:$2b4^{he} 2b5^{ho}$ - placebo vs. 7:$2b4^{he} 2b5^{ho}$ - lithium	0,1542	0,7135	-0,5593	0,07981	4	3	9,91	82
4:WT - lithium vs. 4:$2b4^{he} 2b5^{ho}$ - lithium	0,5385	0,2521	0,2864	0,07587	4	4	5,338	82
4:WT - lithium vs. 5:WT - placebo	0,5385	0,2008	0,3377	0,07587	4	4	6,295	82
4:WT - lithium vs. 5:$2b4^{he} 2b5^{ho}$ - placebo	0,5385	0,1599	0,3785	0,07587	4	4	7,056	82
4:WT - lithium vs. 5:WT - lithium	0,5385	0,929	-0,3906	0,05684	4	4	9,718	70
4:WT - lithium vs. 5:$2b4^{he} 2b5^{ho}$ - lithium	0,5385	0,3769	0,1616	0,07587	4	4	3,011	82
4:WT - lithium vs. 6:WT - placebo	0,5385	0,1552	0,3833	0,07587	4	4	7,144	82
4:WT - lithium vs. 6:$2b4^{he} 2b5^{ho}$ - placebo	0,5385	0,1651	0,3734	0,07587	4	4	6,96	82
4:WT - lithium vs. 6:WT - lithium	0,5385	1,234	-0,6957	0,05684	4	4	17,31	70
4:WT - lithium vs. 6:$2b4^{he} 2b5^{ho}$ - lithium	0,5385	0,4701	0,06833	0,07587	4	4	1,274	82
4:WT - lithium vs. 7:WT - placebo	0,5385	0,192	0,3465	0,07587	4	4	6,459	82
4:WT - lithium vs. 7:$2b4^{he} 2b5^{ho}$ - placebo	0,5385	0,1614	0,3771	0,07587	4	4	7,029	82
4:WT - lithium vs. 7:WT - lithium	0,5385	1,441	-0,9028	0,05684	4	4	22,46	70
4:WT - lithium vs. 7:$2b4^{he} 2b5^{ho}$ - lithium	0,5385	0,7135	-0,175	0,07981	4	3	3,101	82
4:$2b4^{he} 2b5^{ho}$ - lithium vs. 5:WT - placebo	0,2521	0,2008	0,05133	0,07587	4	4	0,9568	82
4:$2b4^{he} 2b5^{ho}$ - lithium vs. 5:$2b4^{he} 2b5^{ho}$ - placebo	0,2521	0,1599	0,09216	0,07587	4	4	1,718	82
4:$2b4^{he} 2b5^{ho}$ - lithium vs. 5:WT - lithium	0,2521	0,929	-0,6769	0,07587	4	4	12,62	82
4:$2b4^{he} 2b5^{ho}$ - lithium vs. 5:$2b4^{he} 2b5^{ho}$ - lithium	0,2521	0,3769	-0,1248	0,05684	4	4	3,106	70
4:$2b4^{he} 2b5^{ho}$ - lithium vs. 6:WT - placebo	0,2521	0,1552	0,09691	0,07587	4	4	1,806	82
4:$2b4^{he} 2b5^{ho}$ - lithium vs. 6:$2b4^{he} 2b5^{ho}$ - placebo	0,2521	0,1651	0,08702	0,07587	4	4	1,622	82
4:$2b4^{he} 2b5^{ho}$ - lithium vs. 6:WT - lithium	0,2521	1,234	-0,982	0,07587	4	4	18,3	82
4:$2b4^{he} 2b5^{ho}$ - lithium vs. 6:$2b4^{he} 2b5^{ho}$ - lithium	0,2521	0,4701	-0,218	0,05684	4	4	5,425	70
4:$2b4^{he} 2b5^{ho}$ - lithium vs. 7:WT - placebo	0,2521	0,192	0,06013	0,07587	4	4	1,121	82
4:$2b4^{he} 2b5^{ho}$ - lithium vs. 7:$2b4^{he} 2b5^{ho}$ - placebo	0,2521	0,1614	0,09071	0,07587	4	4	1,691	82
4:$2b4^{he} 2b5^{ho}$ - lithium vs. 7:WT - lithium	0,2521	1,441	-1,189	0,07587	4	4	22,17	82
4:$2b4^{he} 2b5^{ho}$ - lithium vs. 7:$2b4^{he} 2b5^{ho}$ - lithium	0,2521	0,7135	-0,4614	0,062	4	3	10,52	70
5:WT - placebo vs. 5:$2b4^{he} 2b5^{ho}$ - placebo	0,2008	0,1599	0,04083	0,07587	4	4	0,7611	82
5:WT - placebo vs. 5:WT - lithium	0,2008	0,929	-0,7283	0,07587	4	4	13,57	82
5:WT - placebo vs. 5:$2b4^{he} 2b5^{ho}$ - lithium	0,2008	0,3769	-0,1761	0,07587	4	4	3,283	82
5:WT - placebo vs. 6:WT - placebo	0,2008	0,1552	0,04558	0,05684	4	4	1,134	70
5:WT - placebo vs. 6:$2b4^{he} 2b5^{ho}$ - placebo	0,2008	0,1651	0,03569	0,07587	4	4	0,6652	82
5:WT - placebo vs. 6:WT - lithium	0,2008	1,234	-1,033	0,07587	4	4	19,26	82
5:WT - placebo vs. 6:$2b4^{he} 2b5^{ho}$ - lithium	0,2008	0,4701	-0,2694	0,07587	4	4	5,021	82
5:WT - placebo vs. 7:WT - placebo	0,2008	0,192	0,008793	0,05684	4	4	0,2188	70
5:WT - placebo vs. 7:$2b4^{he} 2b5^{ho}$ - placebo	0,2008	0,1614	0,03937	0,07587	4	4	0,7339	82
5:WT - placebo vs. 7:WT - lithium	0,2008	1,441	-1,24	0,07587	4	4	23,12	82
5:WT - placebo vs. 7:$2b4^{he} 2b5^{ho}$ - lithium	0,2008	0,7135	-0,5127	0,07981	4	3	9,085	82
5:$2b4^{he} 2b5^{ho}$ - placebo vs. 5:WT - lithium	0,1599	0,929	-0,7691	0,07587	4	4	14,34	82
5:$2b4^{he} 2b5^{ho}$ - placebo vs. 5:$2b4^{he} 2b5^{ho}$ - lithium	0,1599	0,3769	-0,217	0,07587	4	4	4,044	82
5:$2b4^{he} 2b5^{ho}$ - placebo vs. 6:WT - placebo	0,1599	0,1552	0,004746	0,07587	4	4	0,08847	82
5:$2b4^{he} 2b5^{ho}$ - placebo vs. 6:$2b4^{he} 2b5^{ho}$ - placebo	0,1599	0,1651	-0,005141	0,05684	4	4	0,1279	70
5:$2b4^{he} 2b5^{ho}$ - placebo vs. 6:WT - lithium	0,1599	1,234	-1,074	0,07587	4	4	20,02	82
5:$2b4^{he} 2b5^{ho}$ - placebo vs. 6:$2b4^{he} 2b5^{ho}$ - lithium	0,1599	0,4701	-0,3102	0,07587	4	4	5,782	82
5:$2b4^{he} 2b5^{ho}$ - placebo vs. 7:WT - placebo	0,1599	0,192	-0,03204	0,07587	4	4	0,5972	82
5:$2b4^{he} 2b5^{ho}$ - placebo vs. 7:$2b4^{he} 2b5^{ho}$ - placebo	0,1599	0,1614	-0,001457	0,05684	4	4	0,03624	70
5:$2b4^{he} 2b5^{ho}$ - placebo vs. 7:WT - lithium	0,1599	1,441	-1,281	0,07587	4	4	23,88	82
5:$2b4^{he} 2b5^{ho}$ - placebo vs. 7:$2b4^{he} 2b5^{ho}$ - lithium	0,1599	0,7135	-0,5535	0,07981	4	3	9,808	82
5:WT - lithium vs. 5:$2b4^{he} 2b5^{ho}$ - lithium	0,929	0,3769	0,5521	0,07587	4	4	10,29	82
5:WT - lithium vs. 6:WT - placebo	0,929	0,1552	0,7738	0,07587	4	4	14,42	82
5:WT - lithium vs. 6:$2b4^{he} 2b5^{ho}$ - placebo	0,929	0,1651	0,764	0,07587	4	4	14,24	82
5:WT - lithium vs. 6:WT - lithium	0,929	1,234	-0,3051	0,05684	4	4	7,592	70
5:WT - lithium vs. 6:$2b4^{he} 2b5^{ho}$ - lithium	0,929	0,4701	0,4589	0,07587	4	4	8,554	82
5:WT - lithium vs. 7:WT - placebo	0,929	0,192	0,7371	0,07587	4	4	13,74	82
5:WT - lithium vs. 7:$2b4^{he} 2b5^{ho}$ - placebo	0,929	0,1614	0,7676	0,07587	4	4	14,31	82
5:WT - lithium vs. 7:WT - lithium	0,929	1,441	-0,5122	0,05684	4	4	12,75	70
5:WT - lithium vs. 7:$2b4^{he} 2b5^{ho}$ - lithium	0,929	0,7135	0,2156	0,07981	4	3	3,82	82
5:$2b4^{he} 2b5^{ho}$ - lithium vs. 6:WT - placebo	0,3769	0,1552	0,2217	0,07587	4	4	4,133	82
5:$2b4^{he} 2b5^{ho}$ - lithium vs. 6:$2b4^{he} 2b5^{ho}$ - placebo	0,3769	0,1651	0,2118	0,07587	4	4	3,949	82
5:$2b4^{he} 2b5^{ho}$ - lithium vs. 6:WT - lithium	0,3769	1,234	-0,8572	0,07587	4	4	15,98	82
5:$2b4^{he} 2b5^{ho}$ - lithium vs. 6:$2b4^{he} 2b5^{ho}$ - lithium	0,3769	0,4701	-0,09322	0,05684	4	4	2,32	70
5:$2b4^{he} 2b5^{ho}$ - lithium vs. 7:WT - placebo	0,3769	0,192	0,1849	0,07587	4	4	3,447	82
5:$2b4^{he} 2b5^{ho}$ - lithium vs. 7:$2b4^{he} 2b5^{ho}$ - placebo	0,3769	0,1614	0,2155	0,07587	4	4	4,017	82
5:$2b4^{he} 2b5^{ho}$ - lithium vs. 7:WT - lithium	0,3769	1,441	-1,064	0,07587	4	4	19,84	82
5:$2b4^{he} 2b5^{ho}$ - lithium vs. 7:$2b4^{he} 2b5^{ho}$ - lithium	0,3769	0,7135	-0,3365	0,062	4	3	7,677	70
6:WT - placebo vs. 6:$2b4^{he} 2b5^{ho}$ - placebo	0,1552	0,1651	-0,009888	0,07587	4	4	0,1843	82
6:WT - placebo vs. 6:WT - lithium	0,1552	1,234	-1,079	0,07587	4	4	20,11	82
6:WT - placebo vs. 6:$2b4^{he} 2b5^{ho}$ - lithium	0,1552	0,4701	-0,3149	0,07587	4	4	5,871	82
6:WT - placebo vs. 7:WT - placebo	0,1552	0,192	-0,03678	0,05684	4	4	0,9153	70
6:WT - placebo vs. 7:$2b4^{he} 2b5^{ho}$ - placebo	0,1552	0,1614	-0,006203	0,07587	4	4	0,1156	82
6:WT - placebo vs. 7:WT - lithium	0,1552	1,441	-1,286	0,07587	4	4	23,97	82
6:WT - placebo vs. 7:$2b4^{he} 2b5^{ho}$ - lithium	0,1552	0,7135	-0,5583	0,07981	4	3	9,892	82
6:$2b4^{he} 2b5^{ho}$ - placebo vs. 6:WT - lithium	0,1651	1,234	-1,069	0,07587	4	4	19,93	82
6:$2b4^{he} 2b5^{ho}$ - placebo vs. 6:$2b4^{he} 2b5^{ho}$ - lithium	0,1651	0,4701	-0,3051	0,07587	4	4	5,686	82
6:$2b4^{he} 2b5^{ho}$ - placebo vs. 7:WT - placebo	0,1651	0,192	-0,0269	0,07587	4	4	0,5013	82
6:$2b4^{he} 2b5^{ho}$ - placebo vs. 7:$2b4^{he} 2b5^{ho}$ - placebo	0,1651	0,1614	0,003685	0,05684	4	4	0,09169	70
6:$2b4^{he} 2b5^{ho}$ - placebo vs. 7:WT - lithium	0,1651	1,441	-1,276	0,07587	4	4	23,79	82
6:$2b4^{he} 2b5^{ho}$ - placebo vs. 7:$2b4^{he} 2b5^{ho}$ - lithium	0,1651	0,7135	-0,5484	0,07981	4	3	9,717	82
6:WT - lithium vs. 6:$2b4^{he} 2b5^{ho}$ - lithium	1,234	0,4701	0,764	0,07587	4	4	14,24	82
6:WT - lithium vs. 7:WT - placebo	1,234	0,192	1,042	0,07587	4	4	19,43	82
6:WT - lithium vs. 7:$2b4^{he} 2b5^{ho}$ - placebo	1,234	0,1614	1,073	0,07587	4	4	20	82
6:WT - lithium vs. 7:WT - lithium	1,234	1,441	-0,2071	0,05684	4	4	5,154	70
6:WT - lithium vs. 7:$2b4^{he} 2b5^{ho}$ - lithium	1,234	0,7135	0,5207	0,07981	4	3	9,226	82
6:$2b4^{he} 2b5^{ho}$ - lithium vs. 7:WT - placebo	0,4701	0,192	0,2782	0,07587	4	4	5,185	82
6:$2b4^{he} 2b5^{ho}$ - lithium vs. 7:$2b4^{he} 2b5^{ho}$ - placebo	0,4701	0,1614	0,3087	0,07587	4	4	5,755	82
6:$2b4^{he} 2b5^{ho}$ - lithium vs. 7:WT - lithium	0,4701	1,441	-0,9711	0,07587	4	4	18,1	82
6:$2b4^{he} 2b5^{ho}$ - lithium vs. 7:$2b4^{he} 2b5^{ho}$ - lithium	0,4701	0,7135	-0,2433	0,062	4	3	5,55	70
7:WT - placebo vs. 7:$2b4^{he} 2b5^{ho}$ - placebo	0,192	0,1614	0,03058	0,07587	4	4	0,57	82
7:WT - placebo vs. 7:WT - lithium	0,192	1,441	-1,249	0,07587	4	4	23,29	82
7:WT - placebo vs. 7:$2b4^{he} 2b5^{ho}$ - lithium	0,192	0,7135	-0,5215	0,07981	4	3	9,24	82
7:$2b4^{he} 2b5^{ho}$ - placebo vs. 7:WT - lithium	0,1614	1,441	-1,28	0,07587	4	4	23,86	82
7:$2b4^{he} 2b5^{ho}$ - placebo vs. 7:$2b4^{he} 2b5^{ho}$ - lithium	0,1614	0,7135	-0,5521	0,07981	4	3	9,782	82
7:WT - lithium vs. 7:$2b4^{he} 2b5^{ho}$ - lithium	1,441	0,7135	0,7278	0,07981	4	3	12,9	82

Figure 4

**Table Analyzed**	**Active b-catenin WT plac vs VWM plac**		**Basic test parameter: WT-VWM differences**		
Unpaired t test					
P value		0,3659			
P value summary	ns				
One- or two-tailed P value?	One-tailed				
t, df	t=0,3626, df=5				
Mean WT		1,196			
Mean VWM		1,138			
95% confidence interval	-0,4623 to 0,3480				
**Table Analyzed**	**Active b-catenin all groups**		**Treatment effects in WT and VWM animals**		
Two-way ANOVA	Ordinary				
Alpha		0,05			
Source of Variation	% of total variation		P value	P value summary	Significant?
Interaction		9,981	0,298	ns	No
Genotype		3,09	0,5549	ns	No
Treatment		3,09	0,5549	ns	No
**Table Analyzed**	**Ephb WT plac vs VWM plac**		**Basic test parameter: WT-VWM differences**		
Mann Whitney test					
P value		0,9497			
Exact or approximate P value?	Exact				
P value summary	ns				
One- or two-tailed P value?	Two-tailed				
Sum of ranks in column A,B	46 , 59				
Mann-Whitney U		23			
Difference between medians					
Median WT	0,03719, n=6				
Median VWM	0,03094, n=8				
Difference: Actual		-0,006254			
Difference: Hodges-Lehmann		-0,002647			
**Table Analyzed**	**Ephb all groups**		**Treatment effects in WT and VWM animals**		
Two-way ANOVA	Ordinary				
Alpha		0,05			
Source of Variation	% of total variation		P value	P value summary	Significant?
Interaction		0,001155	0,9867	ns	No
Genotype		0,008857	0,9633	ns	No
Treatment		1,661	0,5303	ns	No
**Table Analyzed**	**Sox9 WT plac vs VWM plac**		**Basic test parameter: WT-VWM differences**		
Mann Whitney test					
P value		0,2345			
Exact or approximate P value?	Exact				
P value summary	ns				
One- or two-tailed P value?	Two-tailed				
Sum of ranks in column A,B	56 , 80				
Mann-Whitney U		20			
Difference between medians					
Median WT	0,4122, n=8				
Median VWM	0,7566, n=8				
Difference: Actual		0,3444			
Difference: Hodges-Lehmann		0,3887			
**Table Analyzed**	**Sox9 all groups**		**Treatment effects in WT and VWM animals**		
Two-way ANOVA	Ordinary				
Alpha		0,05			
Source of Variation	% of total variation		P value	P value summary	Significant?
Interaction		1,14	0,5542	ns	No
Genotype		8,114	0,1215	ns	No
Treatment		1,661	0,476	ns	No
**Table Analyzed**	**Axin2 WT plac vs VWM plac**		**Basic test parameter: WT-VWM differences**		
Mann Whitney test					
P value		0,7984			
Exact or approximate P value?	Exact				
P value summary	ns				
One- or two-tailed P value?	Two-tailed				
Sum of ranks in column A,B	65 , 71				
Mann-Whitney U		29			
Difference between medians					
Median WT	0,05794, n=8				
Median VWM	0,06222, n=8				
Difference: Actual		0,004273			
Difference: Hodges-Lehmann		0,01266			
**Table Analyzed**	**Axin2 all groups**		**Treatment effects in WT and VWM animals**		
Two-way ANOVA	Ordinary				
Alpha		0,05			
Source of Variation	% of total variation		P value	P value summary	Significant?
Interaction		0,9327	0,6075	ns	No
Genotype		0,1109	0,8591	ns	No
Treatment		2,176	0,4342	ns	No

Figure 5

**Table Analyzed**	**rel. p-eIF2a WT-plac vs VWM-plac**		**Basic test parameter: WT-VWM differences**
Mann Whitney test			
P value	<0,0001		
Exact or approximate P value?	Exact		
P value summary	****		
One- or two-tailed P value?	One-tailed		
Sum of ranks in column A,B	100 , 36		
Mann-Whitney U		0	
Median WT	0,03143, n=8		
Median VWM	0,01099, n=8		
Difference: Actual		-0,02044	
Difference: Hodges-Lehmann		-0,02051	
**Table Analyzed**	**WT treatment rel. p-eIF2a**		**Basic test parameter: Treatment effect in WT animals**
Unpaired t test			
P value		0,3193	
P value summary	ns		
One- or two-tailed P value?	One-tailed		
t, df	t=0,4801, df=14		
Mean of placebo		0,03308	
Mean of lithium		0,03583	
95% confidence interval	-0,009529 to 0,01503		
**Table Analyzed**	**VWM treatment rel. p-eIF2a**		**Treatment effects in VWM animals**
Unpaired t test			
P value		0,1087	
P value summary	ns		
One- or two-tailed P value?	One-tailed		
t, df	t=1,297, df=13		
Mean of placebo		0,01041	
Mean of lithium		0,0126	
95% confidence interval	-0,001459 to 0,005840		
**Table Analyzed**	**Chop WT-plac vs VWM-plac**		**Basic test parameter: WT-VWM differences**
Mann Whitney test			
P value		0,0074	
Exact or approximate P value?	Exact		
P value summary	**		
One- or two-tailed P value?	One-tailed		
Sum of ranks in column A,B	45 , 91		
Mann-Whitney U		9	
Median WT	1,973, n=8		
Median VWM	4,167, n=8		
Difference: Actual		2,194	
Difference: Hodges-Lehmann		2,341	
**Table Analyzed**	**WT treatment Chop**		**Basic test parameter: Treatment effect in WT animals**
Unpaired t test			
P value		0,0071	
P value summary	**		
One- or two-tailed P value?	One-tailed		
t, df	t=2,801, df=14		
Mean placebo		1,914	
Mean lithium		3,838	
95% confidence interval	0,4510 to 3,397		
**Table Analyzed**	**VWM treatment Chop**		**Treatment effects in VWM animals**
Mann Whitney test			
P value		0,1393	
Exact or approximate P value?	Exact		
P value summary	ns		
One- or two-tailed P value?	One-tailed		
Sum of ranks in column A,B	57 , 79		
Mann-Whitney U		21	
Median placebo	4,167, n=8		
Median lithium	8,070, n=8		
Difference: Actual		3,903	
Difference: Hodges-Lehmann		2,372	
**Table Analyzed**	**Eif4epb1 WT-plac vs VWM-plac**		**Basic test parameter: WT-VWM differences**
Mann Whitney test			
P value	<0,0001		
Exact or approximate P value?	Exact		
P value summary	****		
One- or two-tailed P value?	One-tailed		
Sum of ranks in column A,B	36 , 100		
Mann-Whitney U		0	
Median WT	0,07875, n=8		
Median VWM	0,7327, n=8		
Difference: Actual		0,6539	
Difference: Hodges-Lehmann		0,6385	
**Table Analyzed**	**WT treatment Eif4epb1**		**Basic test parameter: Treatment effect in WT animals**
Unpaired t test			
P value		0,0749	
P value summary	ns		
One- or two-tailed P value?	One-tailed		
t, df	t=1,524, df=14		
Mean placebo		0,0879	
Mean lithium		0,1499	
95% confidence interval	-0,02525 to 0,1493		
**Table Analyzed**	**VWM treatment_Eif4epb1**		**Treatment effects in VWM animals**
Unpaired t test			
P value		0,0802	
P value summary	ns		
One- or two-tailed P value?	One-tailed		
t, df	t=1,482, df=14		
Mean placebo		1,532	
Mean lithium		3,226	
95% confidence interval	-0,7568 to 4,145		
**Table Analyzed**	**GADD34 WT-plac vs VWM-plac**		**Basic test parameter: WT-VWM differences**
Mann Whitney test			
P value		0,0141	
Exact or approximate P value?	Exact		
P value summary	*		
One- or two-tailed P value?	One-tailed		
Sum of ranks in column A,B	47 , 89		
Mann-Whitney U		11	
Median WT	0,4045, n=8		
Median VWM	0,7020, n=8		
Difference: Actual		0,2975	
Difference: Hodges-Lehmann		0,2549	
**Table Analyzed**	**WT treatment GADD34**		**Basic test parameter: Treatment effect in WT animals**
Unpaired t test			
P value		0,061	
P value summary	ns		
One- or two-tailed P value?	One-tailed		
t, df	t=1,646, df=14		
Mean placebo		0,5026	
Mean lithium		0,8763	
95% confidence interval	-0,1133 to 0,8608		

Table Analyzed	VWM treatment GADD34		Treatment effects in VWM animals
Mann Whitney test			
P value		0,0035	
Exact or approximate P value?	Exact		
P value summary	**		
One- or two-tailed P value?	One-tailed		
Sum of ranks in column A,B	43 , 93		
Mann-Whitney U		7	
Difference between medians			
Mean placebo	0,7020, n=8		
Mean lithium	1,270, n=8		
Difference: Actual		0,5681	
Difference: Hodges-Lehmann		0,5333	

Table Analyzed	Nupr WT-plac vs VWM-plac		Basic test parameter: WT-VWM differences
Mann Whitney test			
P value	<0,0001		
Exact or approximate P value?	Exact		
P value summary	****		
One- or two-tailed P value?	One-tailed		
Sum of ranks in column A,B	36 , 100		
Mann-Whitney U		0	
Median WT	1,468, n=8		
Median VWM	13,36, n=8		
Difference: Actual		11,9	
Difference: Hodges-Lehmann		11,84	

Table Analyzed	WT treatment Nupr		Basic test parameter: Treatment effect in WT animals
Unpaired t test			
P value		0,0949	
P value summary	ns		
One- or two-tailed P value?	One-tailed		
t, df	t=1,378, df=14		
Mean of placebo		1,642	
Mean of lithium		2,493	
95% confidence interval	-0,4729 to 2,174		

Table Analyzed	VWM treatment Nupr		Treatment effects in VWM animals
Mann Whitney test			
P value		0,0524	
Exact or approximate P value?	Exact		
P value summary	ns		
One- or two-tailed P value?	One-tailed		
Sum of ranks in column A,B	52 , 84		
Mann-Whitney U		16	
Median placebo	13,36, n=8		
Median lithium	24,52, n=8		
Difference: Actual		11,16	
Difference: Hodges-Lehmann		8,917	

Table Analyzed	SLC7a3 WT-plac vs VWM-plac		Basic test parameter: WT-VWM differences
Unpaired t test			
P value	<0,0001		
P value summary	****		
One- or two-tailed P value?	One-tailed		
t, df	t=6,594, df=14		
Mean WT		0,3955	
Mean VWM		1,37	
95% confidence interval	0,6576 to 1,292		

Table Analyzed	WT treatment SLC7a3		Basic test parameter: Treatment effect in WT animals
Unpaired t test			
P value		0,0403	
P value summary	*		
One- or two-tailed P value?	One-tailed		
t, df	t=1,883, df=14		
Mean placebo		0,3955	
Mean lithium		0,4639	
95% confidence interval	-0,009502 to 0,1464		

Table Analyzed	VWM treatment SLC7a3		Treatment effects in VWM animals
Unpaired t test			
P value		0,0041	
P value summary	**		
One- or two-tailed P value?	One-tailed		
t, df	t=3,073, df=14		
Mean placebo		1,37	
Mean lithium		2,414	
95% confidence interval	0,3154 to 1,772		

Supplementary Figure 1

Table Analyzed	Trib3 WT-plac vs VWM-plac		Basic test parameter: WT-VWM differences
Mann Whitney test			
P value		0,0023	
Exact or approximate P value?	Exact		
P value summary	**		
One- or two-tailed P value?	One-tailed		
Sum of ranks in column A,B	42 , 94		
Mann-Whitney U		6	
Median WT	0,05583, n=8		
Median VWM	1,390, n=8		
Difference: Actual		1,334	
Difference: Hodges-Lehmann		1,327	

Table Analyzed	WT treatment Trib3		Basic test parameter: Treatment effect in WT animals
Unpaired t test			
P value		0,1484	
P value summary	ns		
One- or two-tailed P value?	One-tailed		
t, df	t=1,084, df=14		
Mean placebo		1,471	
Mean lithium		2,216	
95% confidence interval	-0,7293 to 2,219		

Table Analyzed	VWM treatment Trib3		Treatment effects in VWM animals
Unpaired t test			
P value		0,1433	
P value summary	ns		
One- or two-tailed P value?	One-tailed		
t, df	t=1,108, df=14		
Mean placebo		0,05908	
Mean lithium		0,09198	
95% confidence interval	-0,03078 to 0,09657		

Table Analyzed	SLC3a2 WT-plac vs VWM-plac		Basic test parameter: WT-VWM differences
Mann Whitney test			
P value		0,0052	
Exact or approximate P value?	Exact		
P value summary	**		
One- or two-tailed P value?	One-tailed		
Sum of ranks in column A,B	44 , 92		
Mann-Whitney U		8	
Median WT	3,059, n=8		
Median VWM	10,21, n=8		
Difference: Actual		7,151	
Difference: Hodges-Lehmann		6,919	

Table Analyzed	WT treatment SLC3a2		Basic test parameter: Treatment effect in WT animals
Unpaired t test			
P value		0,3628	
P value summary	ns		
One- or two-tailed P value?	One-tailed		
t, df	t=0,3582, df=14		
Mean placebo		3,069	
Mean lithium		3,327	
95% confidence interval	-1,288 to 1,804		

Table Analyzed	VWM treatment SLC3a2		Treatment effects in VWM animals
Unpaired t test			
P value		0,2467	
P value summary	ns		
One- or two-tailed P value?	One-tailed		
t, df	t=0,7034, df=14		
Mean placebo		9,715	
Mean lithium		11,23	
95% confidence interval	-3,107 to 6,140		

Table Analyzed	Psat1 WT-plac vs VWM-plac		Basic test parameter: WT-VWM differences
Unpaired t test			
P value		0,009	
P value summary	**		
One- or two-tailed P value?	One-tailed		
t, df	t=2,678, df=14		
Mean WT		3,931	
Mean VWM		8,508	
95% confidence interval	0,9114 to 8,242		

Table Analyzed	WT treatment Psat1		Basic test parameter: Treatment effect in WT animals
Unpaired t test			
P value		0,0937	
P value summary	ns		
One- or two-tailed P value?	One-tailed		
t, df	t=1,386, df=14		
Mean placebo		3,931	
Mean lithium		5,399	
95% confidence interval	-0,8036 to 3,739		

Table Analyzed	VWM treatment Psat1		Treatment effects in VWM animals
Mann Whitney test			
P value		0,3227	
Exact or approximate P value?	Exact		
P value summary	ns		
One- or two-tailed P value?	One-tailed		
Sum of ranks in column A,B	63 , 73		
Mann-Whitney U		27	
Median placebo	8,333, n=8		
Median lithium	9,522, n=8		
Difference: Actual		1,189	
Difference: Hodges-Lehmann		1,342	
