# Supplementary material for: Lithium: effects in animal models of vanishing white matter are not promising
Source: Front Neurosci. 2024 Jan 30;18:1275744. doi: 10.3389/fnins.2024.1275744 (PMC10861708; doi:10.3389/fnins.2024.1275744)
Supplement: SUPPLEMENTARY DATA SHEET 2 — qPCR primers. [file Data_Sheet_2.PDF]

Oligonucleotide primer		Sequence
Sorcs1	5' to 3' (forward)	GGG ACA TCA GCC GAG TCA TC
	3' to 5' (reverse)	AAC ACC GCC ACC AGG ATA TG
Nrxn	5' to 3' (forward)	CCC CAC AAA GGA ACC CAT CA
	3' to 5' (reverse)	GTT GGC TAA CCC ACC TGA G
EphB	5' to 3' (forward)	GAT GCC CGC TAC GTA GTT CA
	3' to 5' (reverse)	CTG GGC ACC TGA ACC TCT TT
Sox9	5' to 3' (forward)	AGG AAG CTG GCA GAC CAG TA
	3' to 5' (reverse)	CTC CTC CAC GAA GGG TCT CT
Axin2	5' to 3' (forward)	GAG AGT GAG CGG CAG AGC
	3' to 5' (reverse)	CGG CTG ACT CGT TCT CCT
Ddit3/Chop	5' to 3' (forward)	CTG GTA TGA GGA TCT GCA GG
	3' to 5' (reverse)	TTG ATT CTT CCT CTT CGT TTC C
Eif4ebp1	5' to 3' (forward)	CAC GCT CTT CAG CAC CAC C
	3' to 5' (reverse)	CCA CAG GTG AGT TCC GAC A
Gadd34	5' to 3' (forward)	GCT CAG ATT GTT CAA AGC CC
	3' to 5' (reverse)	CTT TCT CAG CGA AGT GTA CC
Nupr	5' to 3' (forward)	CTG CTG CCA ATA CCA ACC
	3' to 5' (reverse)	CTG GCC TTA TCT CCA GCT C
Slc7a3	5' to 3' (forward)	AGA GGA GAC ACT TGA AGC AG
	3' to 5' (reverse)	AGT TAG TAG TAC GGC AAG CAG
Trib	5' to 3' (forward)	TGT CTT CAG CAA CTG TGA GAG GAC GAA G
	3' to 5' (reverse)	GTA GGA TGG CCG GGA GCT GAG TAT C
Slc3a2	5' to 3' (forward)	GAA AGC TGA TGA ATG CAC CC
	3' to 5' (reverse)	CAA TTT GCT GCA GGT CAG AG
Psat1	5' to 3' (forward)	GAT GTT TCC AAG TTT GGT GTG
	3' to 5' (reverse)	CCT GCA CTT TGT AGT CAA GG
