# Supplementary figures and images for: Lithium: effects in animal models of vanishing white matter are not promising
Source: Front Neurosci. 2024 Jan 30;18:1275744. doi: 10.3389/fnins.2024.1275744 (PMC10861708; doi:10.3389/fnins.2024.1275744)

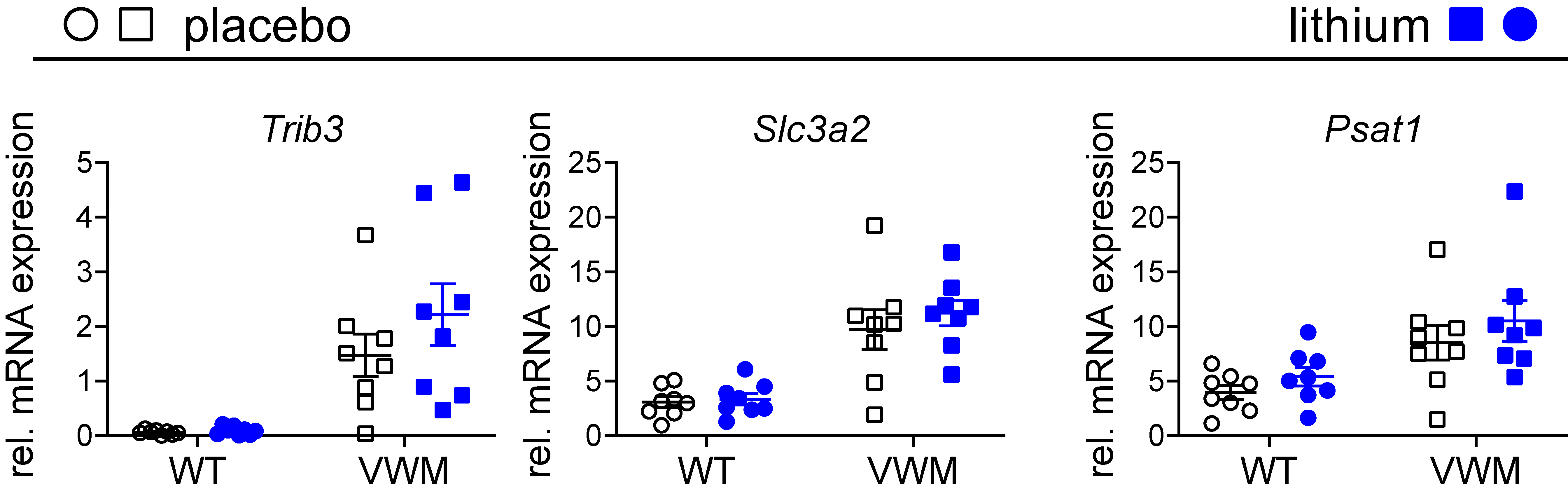

Supplement: SUPPLEMENTARY IMAGE 1 — Subtle increase of the ATF4-regulated transcriptome in the mouse cerebellum by lithium. [file Image_1.TIF]

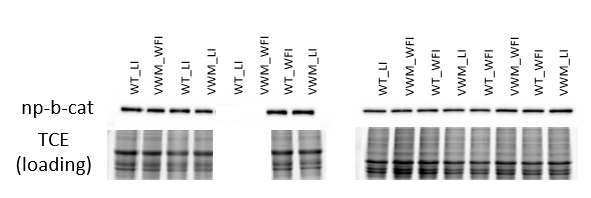

Supplement: SUPPLEMENTARY IMAGE 2 — Overview of gels and immunoblots used for the quantification of non-phosphorylated beta-catenin shown in figure 4. [file Image_2.TIF]

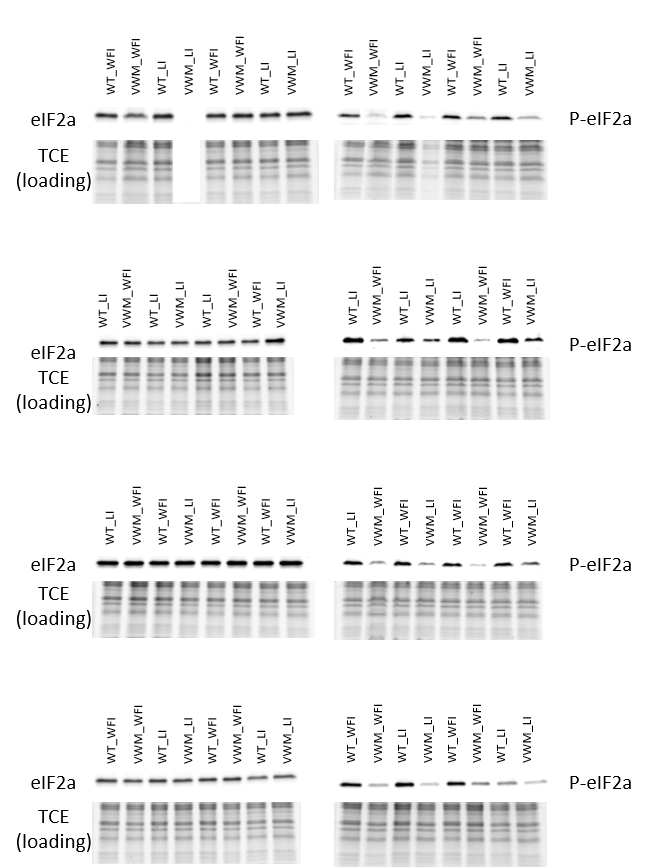

Supplement: SUPPLEMENTARY IMAGE 3 — Overview of gels and immunoblots used for the quantification of eIF2α phosphorylation shown in figure 5. [file Image_3.TIF]
